# Tau mutant A152T, a risk factor for FTD/PSP, induces neuronal dysfunction and reduced lifespan independently of aggregation in a *C. elegans* Tauopathy model

**DOI:** 10.1186/s13024-016-0096-1

**Published:** 2016-04-27

**Authors:** Ghulam Jeelani Pir, Bikash Choudhary, Eckhard Mandelkow, Eva-Maria Mandelkow

**Affiliations:** German Center for Neurodegenerative Diseases (DZNE), Ludwig-Erhard-Allee 2, 53175 Bonn, Germany; Caesar Research Center, Ludwig-Erhard-Allee 2, 53175 Bonn, Germany; Max-Planck-Institute for Metabolism Research (Cologne), Hamburg Outstation, c/o DESY, Notkestrasse 85, 22607 Hamburg, Germany

**Keywords:** AD, Alzheimer disease, PSP, Progressive supranuclear palsy, FTD, Frontotemporal dementia, CBD, Corticobasal degeneration

## Abstract

**Background:**

A certain number of mutations in the Microtubule-Associated Protein Tau (*MAPT*) gene have been identified in individuals with high risk to develop neurodegenerative diseases, collectively called tauopathies. The mutation A152T*MAPT* was recently identified in patients diagnosed with frontotemporal spectrum disorders, including Progressive Supranuclear Palsy (PSP), Frontotemporal Dementia (FTD), Corticobasal Degeneration (CBD), and Alzheimer disease (AD). The A152T*MAPT* mutation is unusual since it lies within the N-terminal region of Tau protein, far outside the repeat domain that is responsible for physiological Tau-microtubule interactions and pathological Tau aggregation. How A152T*MAPT* causes neurodegeneration remains elusive.

**Results:**

To understand the pathological consequences of this mutation, here we present a new *Caenorhabditis elegans* model expressing the mutant A152T*MAPT* in neurons. While expression of full-length wild-type human tau (Tau^wt^, 2N4R) in *C. elegans* neurons induces a progressive mild uncoordinated locomotion in a dose-dependent manner, mutant tau (Tau^A152T^, 2N4R) induces a severe paralysis accompanied by acute neuronal dysfunction. Mutant Tau^A152T^ worms display morphological changes in neurons reminiscent of neuronal aging and a shortened life-span. Moreover, mutant A152T overexpressing neurons show mislocalization of pre-synaptic proteins as well as distorted mitochondrial distribution and trafficking. Strikingly, mutant tau-transgenic worms do not accumulate insoluble tau aggregates, although soluble oligomeric tau was detected. In addition, the full-length A152T-tau remains in a pathological conformation that accounts for its toxicity. Moreover, the N-terminal region of tau is not toxic per se, despite the fact that it harbours the A152T mutation, but requires the C-terminal region including the repeat domain to move into the neuronal processes in order to execute the pathology.

**Conclusion:**

In summary, we show that the mutant Tau^A152T^ induces neuronal dysfunction, morphological alterations in neurons akin to aging phenotype and reduced life-span independently of aggregation. This comprehensive description of the pathology due to Tau^A152T^ opens up multiple possibilities to identify cellular targets involved in the Tau-dependent pathology for a potential therapeutic intervention.

**Electronic supplementary material:**

The online version of this article (doi:10.1186/s13024-016-0096-1) contains supplementary material, which is available to authorized users.

## Background

Neurodegenerative diseases are a leading cause of dementia and pose a serious threat to our aging population. Among them, tauopathies (including AD and FTDP-17) characterized by the accumulation of highly phosphorylated insoluble tau [1–3] are the most common. Tauopathies can be of two types [4]; sporadic and familial. A small percentage of patients (1–5 %) develop disease as a result of mutations in the tau gene (*MAPT*). These mutations can have multifarious consequences; they are capable of reducing the microtubule binding affinity of tau and therefore result in a partial loss of function of tau [5]. Moreover, these mutations are able to increase the beta-propensity of tau, making it more prone to aggregation. Consistent with this, the majority of *MAPT* mutations studied so far are clustered in or near the repeat domain of tau which is responsible for aggregation [1]. However, a rare point mutation in tau gene (A152T) was recently reported in patients suffering from PSP, FTD, or AD [6–8]. This mutation is unique since it lies far outside of the repeat domain in the N-terminal proline-rich region which is thought to interact with different signalling pathways due to its interactions with proteins containing SH3-domains [9, 10].

The amino acid substitution A152T in tau has been shown *in vitro* to be associated with a reduced microtubule binding affinity and, an increase in formation of Tau oligomers instead of insoluble PHFs [6]. Whether this amino acid substitution has any bearing on the development of disease remains uncertain, due to scarcity of *in vivo* data. Therefore, to understand the pathological consequences of the A152T mutation in the context of a whole organism, we used the nematode *C. elegans* as a model. It offers several advantages over other animal models. For example, it has a transparent body with a simple nervous system, which makes it possible to image the progression of pathology at the cellular and sub-cellular level. It has a short life-span (2–3 weeks) and is genetically well characterized and tractable. For these reasons, *C. elegans* has been extensively used as a model of neurodegeneration [11–13] and has provided invaluable insights into the nature of the pathology involved. Several key pathways and signalling molecules are conserved between worms and mammals [14]. Two genes implicated in tau pathology (*sut-1* and *sut-2*) have been identified in a genetic screen using *C. elegans* [15, 16], which point to effects involving the microtubule and actin cytoskeleton. More recently, we demonstrated the potential of using *C. elegans* as a tool for the screening of neuroprotective compounds; in fact one of the compounds (MB) that proved beneficial to this model [17] is running in the 3^rd^ phase of clinical trial [18]. All in all, studies of the *C. elegans* nervous system have greatly aided efforts to analyze the causes of neurodegenerative diseases on the path to developing an effective treatment.

To investigate the functional consequences of this mutation *in vivo*, we generated pan-neuronal mutant A152T-tau (htau40A152T, hereafter referred to Tau^AT^) and wild-type tau (htau40WT, or Tau^wt^) overexpressing nematodes. We found that, while the expression of Tau^wt^ led only to mild pathology over time and in dose-dependent manner, the mutant Tau^AT^ showed pronounced pathology from an early age. The pathology induced by Tau^AT^ was dose-independent, manifested as severe paralysis, defects in the GABAergic motor neurons, accumulation of morphological abnormalities in touch neurons reminiscent of neuronal aging and reduced life span. At the cellular level, Tau^AT^ worms displayed altered distribution of major neuronal organelles (synaptic vesicles and mitochondria) and a perturbed mitochondrial trafficking. The worms expressing Tau^AT^ showed the protein in a pathological state as confirmed by staining with the conformation-dependent MC1 antibody, whereas Tau^wt^ worms showed only minimal staining. Furthermore, in mutant worms Tau^AT^ appeared mostly as soluble higher molecular weight oligomers, albeit they did not accumulate as insoluble aggregates. We also demonstrated that although the mutation site lies in the N-terminal half of Tau^AT^, the C-terminal half which engages with the microtubules is necessary for pathology.

## Results

### Expression of mutant Tau^AT^ leads to severe locomotor defects

**Fig. 1:**
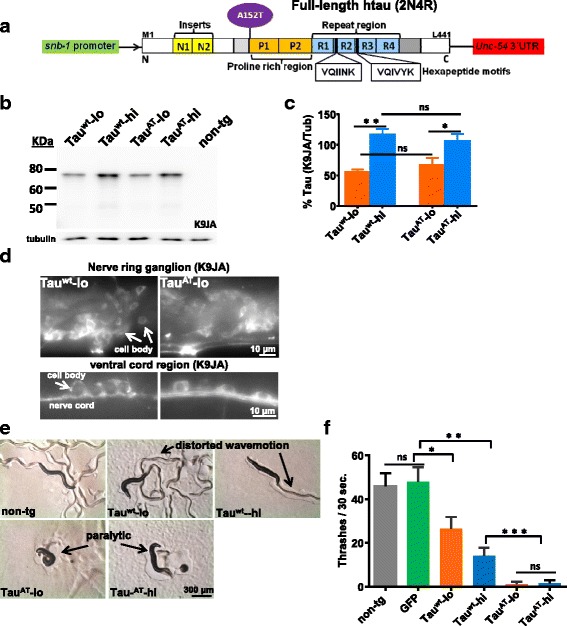
Mutant human Tau^AT^ causes severe paralysis in *C. elegans*. **a** Bar diagram of Tau (2N4R, 441 residues, largest isoform in human CNS) with 2 inserts (N1, N2 near the N-terminus) and 4 repeats (R1-R4 in the C-terminal half) used to generate tau-transgenic lines. The point mutation A152T lies outside of the repeat domain in the proline rich region. The pan-neuronal promoter (*Psnb-1*) drives the expression of human tau cDNA in the *C. elegans* nervous system and the 3′UTR aids in tau expression. **b** Total worm lysates from synchronized day-3 old adults analyzed for Tau by western blotting using pan-tau antibody K9JA. Two independently integrated strains expressing Tau at comparably low and high levels were selected from each transgene: wild-type htau40 (Tau^wt^-lo and Tau^wt^-hi) and mutant htau40A152T (Tau^AT^-lo and Tau^AT^-hi). Tubulin serves as internal control. **c** Quantification of total tau in wild-type Tau lines (Tau^wt^-lo and Tau^wt^-hi) and mutant Tau-A152T lines (Tau^AT^-lo and Tau^AT^-hi). Error bars denote SEM. One-way ANOVA with Tukey’s test was applied for multiple comparisons (ns, non-significant, *P < 0.05, **P < 0.01). **d** Immunochemistry of whole worms with pan-tau K9JA antibody shows tau staining in the nervous system. Top panels depict nerve ring ganglion, bottom panels show ventral cord region. **e** Synchronized day-1 old adults were placed onto the centre of NGM plates freshly spotted with *E. coli* OP-50 and photographed after 10 min. Both Tau^AT^-lo and Tau^AT^-hi worms show strong paralytic phenotype apparent by their coiled body shape and lack of tracks on the *E. coli* lawn. The corresponding lines expressing wild-type human tau (Tau^wt^-lo and Tau^wt^-hi) possess a mild uncoordinated phenotype and are able to crawl away from the point of origin. **f** Thrashing assay of synchronized day-1 old adults in liquid. Control worms (non-tg control or worms expressing GFP in the pharynx as transformation marker only) display a high frequency of ~50 thrashes/30s. Worms expressing Tau^wt^ display a dose-dependent motor impairment with reduced thrashing frequency (~30/30s for Tau^wt^ -lo, ~20/30s for Tau^wt^ -hi). Motor impairment is very severe in the worms expressing mutant Tau^AT^ (both -lo and -hi, ~2/30s). One-way ANOVA with Tukey’s test was applied for multiple comparisons. Error bars denote SEM, n *≥* 30. (ns., non-significant, *P < 0.05, **P < 0.01, ***P < 0.001)

To examine the consequences of the Tau mutation A152T *in vivo*, we generated transgenic worms expressing constitutively either the wild-type Tau^wt^ or the Tau^AT^ mutant form of human 2N4R Tau in neurons. The pan-neuronal expression was achieved using the synaptobrevin promoter *snb-1*p (Fig. 1a). Multiple lines were obtained after transgene integration. We first compared the protein levels by subjecting the worm lysates from each strain (3 day old adults) to western blotting using K9JA pan-Tau antibody. Two lines from each set of transgenes were selected for further analysis; worms showing comparably lower expression levels (referred to hereafter as Tau^wt^-lo or Tau^AT^-lo, resp.) or higher expression levels (termed Tau^wt^-hi and Tau^AT^-hi, resp.). Total tau levels in Tau^wt^-lo and Tau^wt^-hi lines were comparable with their mutant counterparts Tau^AT^-lo and Tau^AT^-hi, respectively (Fig. 1b, c). Immunostaining with K9JA antibody showed that Tau transgenes were expressed in the entire nervous system, notably in the major ganglion nerve ring and the nerve cords (Fig. 1d, Additional file 9). Non-transgenic animals did not show any staining for human Tau (data not shown).

Worms expressing Tau^wt^ exhibit motor defects with mild uncoordinated (Unc) phenotype characterized by sluggishness and a distorted sinusoidal locomotion relative to non-transgenic worms (Fig. 1e). However, in worms expressing Tau^AT^ the severe Unc phenotype is much more pronounced than in worms expressing Tau^wt^. Most of the worms expressing mutant Tau^AT^ were paralytic and were often seen in the coiled state, almost completely unable to move (Fig. 1e). The extent of motor dysfunction in worms expressing Tau^wt^ correlated with the protein expression levels, Tau^wt^-lo with lower tau levels showed a milder phenotype (~30 thrashes/30 s) than Tau^wt^-hi line (~20 thrashes/30 s) with higher tau levels (Fig. 1f, also see Additional file 1: Movie S1 and Additional file 2: Movie S2). By contrast, Tau^AT^-induced toxicity did not correlate with protein expression levels; the Unc phenotype was equally severe in Tau^AT^-lo and Tau^AT^-hi worms (Fig. 1f, also see Additional file 3: Movie S3 and Additional file 4: Movie S4). Tau transgenes (Tau^wt^ and Tau^AT^) when expressed as extrachromosomal arrays showed comparable Tau levels as detected by western blot using K9JA antibody (see Additional file 10), and yielded similar phenotypes as the integrated lines (see Additional file 10), ruling out any effects due to unwanted background mutations during integration.

### Mutant Tau^AT^ leads to pronounced GABAergic neurodegeneration, while Tau^wt^ causes mild degeneration only at higher levels

**Fig. 2:**
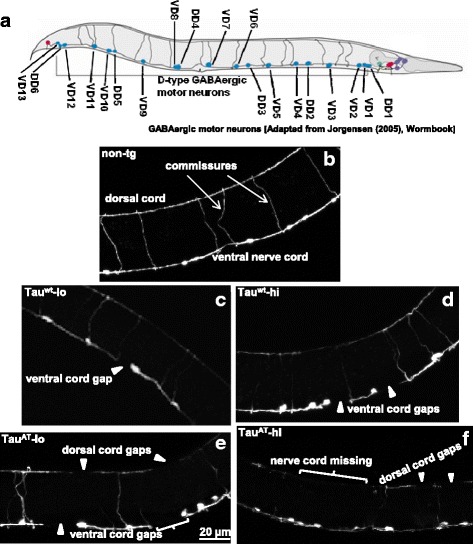
Mutant Tau^AT^ leads to substantial damage in GABAergic motor neurons. *Punc-25::gfp* reporter that labels GABAergic inhibitory neurons with GFP, was crossed with respective tau-transgenic worms to visualize these neurons. **a** Cartoon depicting the GABAergic inhibitory motor system in an adult worm. **b-f** depict representative maximum intensity projections (MIP) of day-1 old adults of the transgenes. For whole worm MIPs, see Additional file 11. Abnormalities seen as gaps in the GABAergic inhibitory neurons are highlighted by arrowheads. GABAergic neurons show normal connectivity in non-tg reporter worms, both dorsal and ventral nerve cords are intact (**b**). Expression of Tau^wt^ produces dose dependent abnormalities in GABAergic neurons, with Tau^wt^ -hi neurons (**d**) accumulating more damage than Tau^wt^ -lo (**c**). However, mutant Tau^AT^ expression leads to severe abnormalities in the form of gaps in the dorsal and ventral nerve cords, both Tau^AT^-lo (**e**) and Tau^AT^-hi (**f**). One of the striking features of mutant Tau^AT^ worms is the absence of stretches of dorsal cord (bracketed areas), not found in non-tg worms or Tau^wt^ worms. See Table 1 for detailed quantitative analysis

Normal sinusoidal locomotion in *C. elegans* is made possible by coordinated activity of reciprocal motor circuits comprised of inhibitory GABAergic and excitatory cholinergic neurons located along the body wall [19], and any interruption in the networking produces a defective locomotion (uncoordinated). To evaluate the possibility that the locomotion phenotype in tau-transgenic worms is due to a defective motor nervous system, the integrity of GABAergic motor system was monitored. Animals were analyzed for axonal gaps in the ventral and dorsal cords by crossing them into the *juIs73*:[*Punc-25::gfp*]III transgene [20] that allows visualization of GABAergic motor neurons by expression of GFP (Fig. 2b). GABAergic motor neurons in both the worm strains expressing Tau^AT^ (Tau^AT^-lo and Tau^AT^-hi) were severely abnormal with frequent gaps in ventral and dorsal cords, often showing stretches of nerve cords missing (Fig. 2e, F and see Additional file 10, Additional file 11), consistent with the extent of motor dysfunction shown by these worms (see Fig. 1e). On the other hand, GABAergic motor neurons in worms expressing Tau^wt^ were slightly affected (lesser gaps) when compared to worms expressing Tau^AT^ (Fig. 2c, d; Table 1). However, when compared to non-tg reporter strain (*juIs73*), Tau^wt^ worms did exhibit a significant number of gaps (Table 1). Consistent with the motor deficits, the extent of neuronal damage seen in worms expressing Tau^wt^ depended on the level of protein expression, with Tau^wt^-hi accumulating more damage than Tau^wt^-lo (Table 1).

**Table 1:** Quantification of GABAergic neuronal damage in day-1 old Tau-tg worms

Strain	VC gaps	DC gaps	N
non-tg	0.4 ± 0.08	0.2 ± 0.14	25
Tau^wt^-lo	1.4 ± 0.17^*^	1.1 ± 0.09	20
Tau^wt^-hi	2.6 ± 0.34^**^	1.7 ± 0.71^*^	18
Tau^AT^-lo	5.8 ± 1.61^***^	6.1 ± 1.14^***^	24
Tau^AT^-hi	6.1 ± 0.98^***^	5.5 ± 1.4^***^	25

Morphological abnormalities in GABAergic inhibitory neurons apparent as gaps in the dorsal cord (DC gaps) and ventral nerve cord (VC gaps) were assessed with the *Punc-25::gfp* reporter. N denotes the total number of animals scored per strain. Non-tg worms with *Punc-25::gfp* reporter serve as controls. *P* values for statistical significance are derived using ANOVA (Kruskal-Wallis test) (*P < 0.05 vs non-tg, **P < 0.01 vs non-tg, ***P < 0.001 vs non-tg)

Since the lines Tau^AT^-lo and Tau^AT^-hi did not differ much in their phenotypes, from hereon we decided to compare the two wild-type tau lines (Tau^wt^-lo and Tau^wt^-hi, which differ depending on Tau levels) with the mutant line with lower tau expression (Tau^AT^-lo), unless otherwise mentioned.

### Mutant Tau^AT^ induces hallmarks of aging neurons and shortens the life-span

**Fig. 3:**
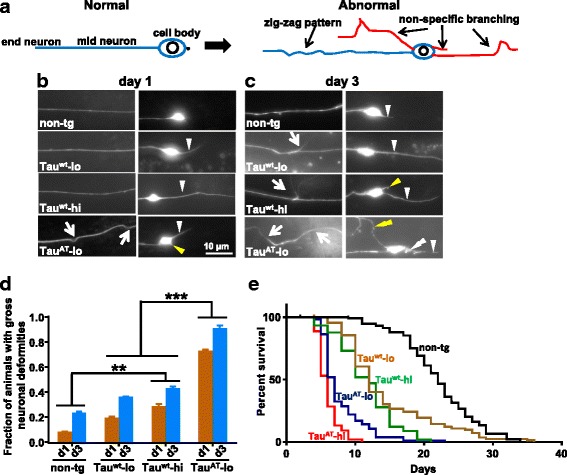
Mutant Tau^AT^ induces morphological changes in mechanosensory neurons early in the adulthood and reduces life-span. **a** Cartoon depicting a normal healthy neuron and a neuron showing morphological changes associated with aging like sprouting, non-specific branching and bending. **b** Mutant Tau^AT^ worms show neuronal abnormalities reminiscent of aging neurons. *Pmec-4::gfp* reporter that expresses GFP in mechanosensory neurons, was crossed into tau-transgenic worms and neurons visualized for morphological abnormalities at the days indicated. Note the soma outgrowth (yellow arrowhead) and bending of neuronal process (white arrow) in Tau^AT^-lo at day 1. At this age, Tau^wt^ (both -lo and -hi) show normal morphology and do not differ from non-tg. Only the incidence of an infrequent posterior extension (white arrow heads) increases in the Tau^wt^ worms (to 30 % compared with 10 % in non-tg at day 1), whereas in Tau^AT^-lo this posterior extension occurs in almost 100 % of the animals. **c** The severity of the phenotype increases with age. Soma outgrowths visible in Tau^AT^-lo animals at day 1 grow and undergo further branching with age (yellow double arrowhead). The volume marker GFP accumulates in beaded structures in the posterior extension in Tau^AT^-lo worms and small outgrowths emanate from posterior extension (white double arrowhead). These beaded structures could represent starting points of new branches, and were previously shown to be associated with mitochondria [24]. **d** Quantification of animals with gross non-specific neuronal abnormalities (bends and branches) at two time points, day 1 and day 3. Error bars denote mean ± SEM, n ≥ 20. **P ≤ 0.01, ***P ≤ 0.001. Paired t-test with unequal variance was used for comparison. **e** Representative survival curves of tau-transgenic animals, non-tg serves as control. Mantel-Cox log-rank test was performed to determine the statistical significance for the worm life-span. (for *P*-values see Table 2)

The above microscopical observations of the GABAergic neurons clearly showed that their processes are degenerating, consistent with the locomotor defects seen in Tau-tg worms. As neuronal restructuring and synaptic disintegration is one of the hallmarks of aging nervous system [21, 22], we asked whether the Tau^AT^ variant initiates such changes which could be interpreted as indication of premature aging of the nervous system. Mechanosensory neurons (touch neurons) offer an excellent model to study changes associated with neuronal aging in *C. elegans*. These neurons undergo morphological changes such as irregular outgrowths from somata, extra neurites, and bending of main neuronal processes that become exaggerated with age [23, 24]. We therefore generated Tau-tg worms carrying *zdIs5:*[*Pmec-4::GFP + lin-15(+)*] transgene that specifically express GFP in the six mechanosensory neurons. We observed significant morphological alterations of these neurons early in adulthood (day 1) of Tau^AT^ worms, changes that have been associated with aging in previous studies [23, 24]. We focussed on ALM neurons (anterior lateral microtubule cells) located in the anterior body region. ALM neuronal processes in Tau^AT^ worms appeared wavy, in contrast to the straight processes seen in non-tg (*zdIs5*) and Tau^wt^ worms (both Tau^wt^-lo and Tau^wt^-hi), and new outgrowths started emanating from the somata already at day 1 (Fig. 3b, panel day1). A higher percentage of Tau^AT^-lo worms (~70 %) showed these morphological abnormalities at day 1 compared to non-tg (~12 %), Tau^wt^-lo (~20 %) and Tau^wt^-hi worms (~23 %) (Fig. 3d). Furthermore, nearly 10 % of non-tg animals showed a single posterior projecting neurite emanating from ALM somata at day 1 consistent with the earlier studies [24], whereas the incidence of such neurites in Tau^AT^-lo ALM neurons was found in almost all the animals. By contrast, worms expressing Tau^wt^ (Tau^wt^-lo and Tau^wt^-hi) showed normal morphology (Fig. 3b, panel day1). However, the number of animals showing ALM neurons with a single posterior projecting neurite was higher in Tau^wt^ worms (~30 %) compared to non-tg animals (~12 %). The severity of these morphological abnormalities further increased with age, new outgrowths that started emanating from ALM somata of day-1 old Tau^AT^-lo worms had extended to a considerable length and occasionally underwent branching. Furthermore, the single posterior projecting neurite also showed new outgrowths at multiple sites by day 3 of adulthood (example in Fig. 3c, panel day 3). By this age, about 30 % of the worms expressing Tau^wt^ showed morphological abnormalities similar to day-1 Tau^AT^ worms (Fig. 3d).

A similar trend was seen in PLM neurons (posterior lateral microtubule cells), but the degree of restructuring (accumulated damage) was less extensive (Tau^wt^-lo: ~10 %, Tau^wt^-hi ~20 % and Tau^AT^-lo: ~60 % at day 1). This is consistent with earlier reports whereby neuronal populations were found to differ in terms of vulnerability during aging [24]. Overall our data suggests that the appearance of these morphological changes in neurons that accompany the aging of *C. elegans* occur early in Tau^AT^ worms compared to Tau^wt^ and non-tg. As previous studies have underlined the role nervous system plays in modulating the lifespan in *C. elegans* [25–29], one would expect a reduced life-span in the worms expressing Tau^AT^. Ultimately, we assessed the survival of Tau-tg animals (Fig. 3e): Tau^wt^ expression resulted in a shorter life-span (~45 % shorter vs. non-tg animals), but mutant Tau^AT^ expression decreased the life-span even much more (~65 % shorter vs. non-tg, ~25 % shorter vs. Tau^wt^ (-lo or -hi) (Table 2).

**Table 2:** Statistical analysis of life-span assay performed as in Fig. 3e legend

Strain	Median survival	# deaths/total N	*P* value (vs non-tg)	*P* value (vs Tau^wt^-lo)	*P* value (vs Tau^wt^-hi)
non-tg	22	111/120		>10^-4^	>10^-4^
Tau^wt^-lo	12	87/120	>10^-4^		>10^-4^
Tau^wt^-hi	12	67/120	>10^-4^	>10^-4^	
Tau^AT^-lo	7	111/120	>10^-4^	>10^-4^	>10^-4^
Tau^AT^-hi	6	110/120	>10^-4^	>10^-4^	>10^-4^

### Mutant Tau^AT^ worms display perturbed distribution of major neuronal organelles and defective presynaptic neurotransmission

**Fig. 4:**
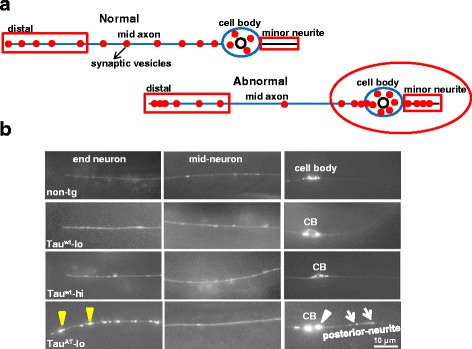
Mutant Tau^AT^ worms show aberrant localization of presynaptic components in mechanosensory neurons. **a** Schematic representation of presynaptic cargo distribution in a normal healthy mechanosensory neuron and a neuron in an aged animal. **b** Day-1 old worms visualized after crossing them into *vdEx262:*[*Pmec-4::mCherry::rab-3*] transgene that expresses mCherry fused to synaptic vesicle associated RAB-3 in mechanosensory neurons. Tau^wt^-lo and Tau^wt^-hi show a similar distribution as non-tg reporter strain. Tau^AT^-lo worms show accumulation of mCherry::RAB-3 in the end neuron (yellow arrowhead), cell body (CB, white arrowhead) and posterior neurite (white arrow). By contrast, the mid-neuron of Tau^AT^-lo shows less puncta (6 ± 3 measured per 40 μm length) than non-tg (15 ± 5) and Tau^wt^-lo (17 ± 6) worms. On day 3, mislocalization of mCherry::RAB-3 worsens in Tau^AT^-lo worms, whereas Tau^wt^-lo and Tau^wt^-hi start accumulating mCherry:RAB-3 puncta in the distal axon and posterior neurite (see Additional file 12)

Since Tau^AT^ worms showed early morphological alterations mimicking aging neuronal phenotypes, we sought to investigate the status of the neuronal transport machinery in the transgenic animals. We checked the distribution of major neuronal cargoes in neurons, which represents an indirect readout for the transport machinery [30]. We first looked at RAB-3, a synaptic vesicle associated Rab GTPase [31], using the the transgene *vdEx262:*[*Pmec-4::mCherry::rab-3;Punc122::gfp*] [32] that expresses mCherry fused to RAB-3 in mechanosensory neurons. Worms expressing Tau^wt^ (Tau^wt^-lo or Tau^wt^-hi) showed uniform distribution of RAB-3 along axons similar to non-tg (*vdEx262*) animals at day 1 (Fig. 4b). In contrast, Tau^AT^-lo worms showed accumulation of RAB-3 in the distal axon and less RAB-3 puncta in the mid-neuron. Furthermore, posterior minor neurites which are usually devoid of RAB-3 (as seen in non-tg worms, Tau^wt^ -lo and Tau^wt^ -hi worms), were abnormally filled with RAB-3 clusters in Tau^AT^-lo worms (Fig. 4b). In older animals, the RAB-3 marker started mislocalizing even in Tau^wt^ worms such that at day 3, RAB-3 was present in minor neurites, similar to day-1 old Tau^AT^-lo worms (Additional file 12). We quantified the percentage of animals with mislocalized mCherry::RAB-3 puncta in the distal axon and the minor neurite at day 1 and day 3. A higher percentage of Tau^AT^-lo animals (~60 %) presented mislocalized mCherry::RAB-3 compared to non-tg worms (~5 %), Tau^wt^-lo (~10 %) and Tau^wt^ -hi (~15 %) worms at day 1. All of these fractions increased substantially with time, with Tau^AT^-lo animals showing the most advanced mislocalization of mCherry::RAB-3 puncta (see Additional file 12 for an example).

**Fig. 5:**
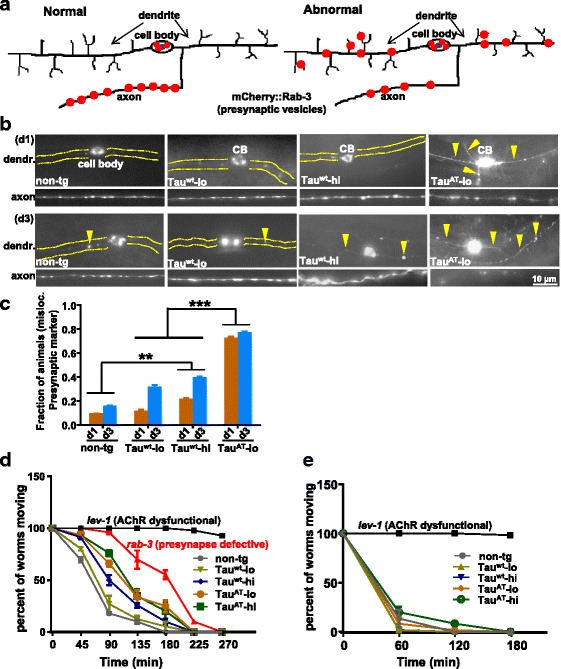
Mutant Tau^AT^ worms show presynaptic protein mislocalization in dendrites of PVD neurons and neurotransmission defects. **a** Schematic representation of a PVD neuron with normal localization of mCherry::RAB-3 (pre-synaptic marker, red dots) and a PVD neuron with altered localization. The branched morphology represents the dendritic compartment; the unbranched ventral process is the axon. **b** Representative images of dendrites and axons at day 1 and day 3 of the mentioned transgenes. In non-tg and Tau^wt^ worms (-lo and -hi), mCherry::RAB-3 localizes in the axonal compartment and is excluded from the dendrites in young adults (day 1). By contrast, Tau^AT^-lo worms show mCherry::RAB-3 mislocalized to the dendrites (yellow arrowheads) and a reduced distribution in the axons. With age, Tau^wt^ (-lo and -hi) also show slight mislocalization to the dendritic compartments (yellow arrowheads). Yellow dotted areas correspond to the dendrite not visible in non-tg, Tau^wt^-lo and Tau^wt^-hi worms at day1. **c** Quantification of the fractions of animals with mislocalized presynaptic mCherry::RAB-3 puncta in tau-transgenes at day 1 (d1) and day 3 (d3). Non-tg worms serve as control. Error bars denote mean ± SEM, n ≥ 20. **P ≤ 0.01, ***P ≤ 0.001. Paired t-test with unequal variance was used for comparison. **d** Time-dependent paralysis induced by aldicarb (acetylcholine esterase inhibitor). Data represents the percentage of worms (mean ± SEM) still able to move on 1 mM aldicarb plates after being touched, as a function of time. Non-tg and Tau^wt^-lo worms are highly sensitive (bottom curves, grey and olive). Tau^wt^-hi, Tau^AT^-lo and Tau^AT^-hi worms show some resistance (blue, ochre, green curves, resp.). Strongly resistant *lev-1* (AChR mutant carrying the *e211* allele) and mildly resistant *rab-3* (Ras GTPase mutant carrying the *js49* allele) are used as additional controls (black and red curve, resp). After applying two-way ANOVA with Bonferroni correction, *P < 0.01* at time points 45 and 180 min, whereas *P < 0.001* at time points 90 and 135 min is obtained for Tau^wt^-hi, Tau^AT^-lo and Tau^AT^-hi against non-tg. *n* = 20 animals, three independent repetitions. **e** Time-dependent paralysis induced by levamisole (acetylcholine receptor agonist). Data represents the percentage of worms (mean ± SEM) still able to move on 0.2 mM levamisole plates after being touched, as a function of time. All the four tau-lines were as sensitive to levamisole as non-tg. Strongly resistant *lev-1 (e211)* is shown as an additional control. *n* = 20 animals, three independent repetitions

We sought to clarify whether the mislocalization of mCherry::RAB-3 is due to a disruption in the polarized protein trafficking. To this end, PVD sensory neurons in *C. elegans* represent suitable test cells because they possess distinct axonal-dendritic compartments, with highly branched dendrites encircling the body, while the axon protrudes to the ventral nerve cord and then grows anteriorly [33, 34] (see schematic in Fig. 5a). We generated Tau-tg worms carrying *kyIs445:Is:*[*Pdes-2::mCherry::RAB-3;des-2::SAD-1::GFP;odr-1::DsRED*] transgene that expresses mCherry fused to RAB-3 in PVD sensory neurons [35]. mCherry::RAB-3 puncta were barely detectable in the PVD dendrites of Tau^wt^ (-lo or -hi) animals, similar to non-tg (*kyIs445*) worms, with only faint signals detectable in Tau^wt^-hi at day 1 and increasing with age (Fig. 5b, c). Contrary to this, in Tau^AT^ worms mCherry::RAB-3 was already mislocalized to the entire PVD dendritic compartment at day 1 (Fig. 5b, panel day1 dendritic). At the same time, the axons of Tau^AT^ worms showed less dense puncta (Fig. 5b, panel day1 axonal). Altered synaptic vesicle distribution could mean that the axonal compartments are compromised in Tau^AT^ worms, implying that there might be a neurotransmission failure at the synapses.

To confirm this, we turned to a complementary approach by using chemicals aldicarb and levamisole that interfere with normal neurotransmission [36]. Aldicarb causes paralysis due to hypercontraction of muscles by inhibiting acetylcholine esterase. Levamisole has a similar effect as an acetylcholine receptor agonist. Normal worms with intact pre- and post-synaptic compartments are sensitive to both chemicals. Worms with either a pre- or a post-synaptic defect show resistance to aldicarb, whereas only worms with a post-synaptic defect show resistance to levamisole. Tau^wt^-lo and non-tg (N2) worms were sensitive to aldicarb, indicating a normal release of acetylcholine at synapses. However, Tau^wt^-hi, Tau^AT^-lo and Tau^AT^-hi showed a mild resistance to aldicarb (Fig. 5d). On the other hand, all the tau-transgenic lines were equally sensitive to levamisole and did not differ from non-tg (Fig. 5e). Thus these experiments argue that the pre-synaptic compartments are compromised in tau-tg worms.

**Fig. 6:**
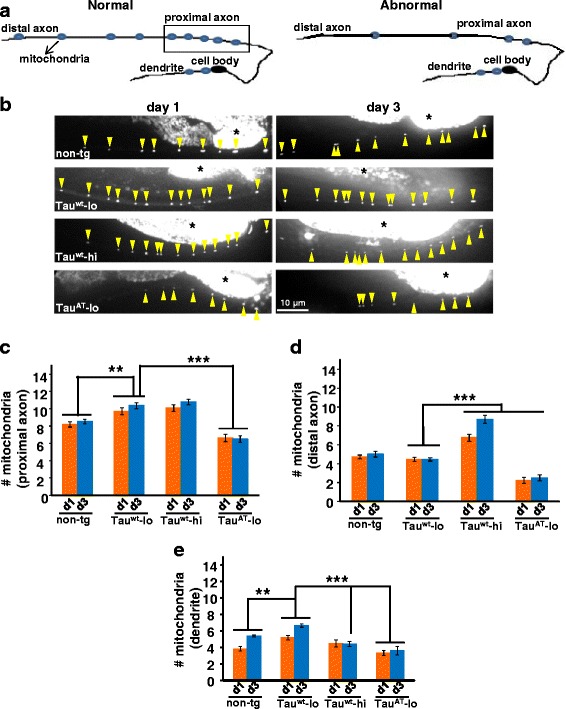
Mutant Tau^AT^ worms display abnormal distribution of mitochondria. **a** Schematic representation of DA9 motor neuron displaying a normal and an abnormal patterning of mitochondria in different compartments (blue dots). **b** Day-1 old adult worms of the tau-transgenes (left panel) visualized after crossing them into the *wyEx2709* [*Pitr-1::TOM-20* ^*1-54aa*^ *::yfp*] reporter that highlights YFP-tagged mitochondria in DA9 motor neurons. Magnified images of the region corresponding to the proximal axon (boxed area in the schematic) are shown to visualize the faint mitochondrial particles (yellow arrowheads). Mitochondrial particles in day-3 old adult worms of the transgenes (right panel). Areas in white marked by asterisk shows body autofluorescence. **c, d,** and **e** Quantification of the average number of mitochondria in different regions of DA9 neurons designated as proximal axon (boxed region), distal axon (away from boxed region) and dendrite. Tau^AT^-lo animals exhibit fewer mitochondria than non-tg and Tau^wt^ worms in all the specified compartments of the neuron at day 1 and day 3. Error bars show mean ± SEM, n ≥ *2*0. **P ≤ 0.01, ***P ≤ 0.001. Paired t-test with unequal variance was used for comparison

Next we analyzed the distribution of another intracellular organelle, mitochondria. The *wyEx2709* [*Pitr-1::TOM-20*^*1-54aa*^*::yfp*] is a transgene that expresses YFP-tagged mitochondria in DA9 motor neurons [37]. The DA9 soma lies in the preanal ganglion; it has a well defined dendrite extending anterioventrally and an axon that crosses the body to reach the dorsal cord and then runs anteriodorsally. Non-tg animals display a typical distribution of labelled mitochondria (schematic in Fig. 6a, [37]; such that the anteriodorsal region (boxed area, ~90 μm, Fig. 6a) contains closely spaced (~5–10 μm) mitochondria, as seen in the non-tg reporter strain (*wyEx2709*) (Fig. 6b, panel non-tg). But away from the anteriodorsal region towards the distal end, the spacing between the mitochondrial particles increases (~30–50 μm) (data not shown). We then visualized mitochondria in our tau-tg animals by crossing them into *wyEx2709*. Tau^AT^-lo worms presented a smaller number of mitochondria in all the three classified compartments of DA9 neurons compared to non-tg, Tau^wt^-lo and Tau^wt^-hi at both the time points (day 1 and day 3) (Fig. 6b). However, compared to non-tg animals, Tau^wt^ expression led to an increase in mitochondria in a dose dependent manner. This effect was more pronounced in the distal axon, where Tau^wt^-hi worms showed ~1.7 fold increase in mitochondrial number compared to Tau^wt^-lo (Fig. 6c, d, e). Collectively, these results show that the mutant Tau^AT^ distorts the normal distribution of neuronal cargoes in *C. elegans* neurons.

### Altered mitochondrial distribution in mutant Tau^AT^ worms is due to a defective transport

**Fig. 7:**
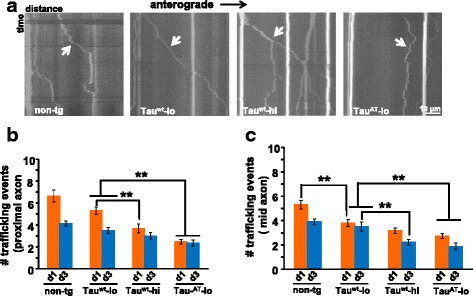
Time-resolved imaging of mitochondria reveals deficits in the trafficking machinery of mutant Tau^AT^ animals. **a** Representative kymographs showing the nature of particle movements in different worm lines. Mitochondria in non-tg, Tau^wt^-lo and Tau^wt^-hi worms show long range movements (in the range 8–15 μm) compared to Tau^AT^-lo where mitochondria exhibit short and oscillatory movements (<5 μm). Quantitation of number of mitochondrial trafficking events in proximal (**b**) and mid region (**c**) of mechanosensory neuron in day 1 and day 3 adults. Tau^AT^-lo animals show significant reduction in mitochondrial trafficking compared to non-tg and Tau^wt^-lo both at day 1 and day 3. However, Tau^wt^ showed dose dependent decreasing trends of trafficking events in both the regions of the neuron. Error bars show mean ± SEM, n ≥ 10. **P ≤ 0.001. Paired t-test with unequal variance was used for comparison

Altered steady state distribution of major neuronal cargoes (synaptic vesicles and mitochondria) in Tau^AT^ animals is likely due to impaired neuronal transport machinery. To investigate the effect of Tau^AT^, we performed mitochondrial trafficking analysis by generating Tau-tg worms carrying *jsIs609* transgene [17] which expresses GFP fused to a mitochondrial localization signal (MLS) in mechanosensory neurons. In order to get an insight into how the transport machinery is affected in different parts of neurons, we chose two regions, one close to the cell body (up to ~80 μm from the cell body) and another in the mid region (120 μm away from the cell body) (refer to Fig. 4a), and recorded mitochondrial movements in a time frame of 2.5 min. The transport behavior was quite different in Tau^AT^-lo compared to non-tg (*jsIs609*) and Tau^wt^ animals. Non-tg and Tau^wt^ animals generally showed long-haul uninterrupted movements (range 10–15 μm), whereas Tau^AT^-lo animals displayed short bidirectional movements (<5 μm) (Fig. 7a, also see Additional file 5: Movie S5, Additional file 6: Movie S6, Additional file 7: Movie S7 and Additional file 8: Movie S8). Quantification revealed a drastic decrease in the number of events (~30 to 60 % reduction) in Tau^AT^-lo animals compared to non-tg and Tau^wt^ (-lo and -hi) animals at both time points. With age, non-tg and Tau^wt^-lo worms showed a decrease in the number of trafficking events, especially in the proximal region (~30–35 % reduction), whereas Tau^wt^ -hi and Tau^AT^-lo did not display a drastic change in the trafficking events in either of the regions with age (Fig. 7b, c). In conclusion, Tau^AT^-lo worms showed a severe defect in mitochondrial transport compared to non-tg and Tau^wt^ animals, which provides a plausible explanation for the altered steady state distribution of neuronal cargoes in the worms expressing mutant Tau^AT^.

### Mutant Tau^AT^ adopts a pathological conformation but does not form insoluble aggregates

**Fig. 8:**
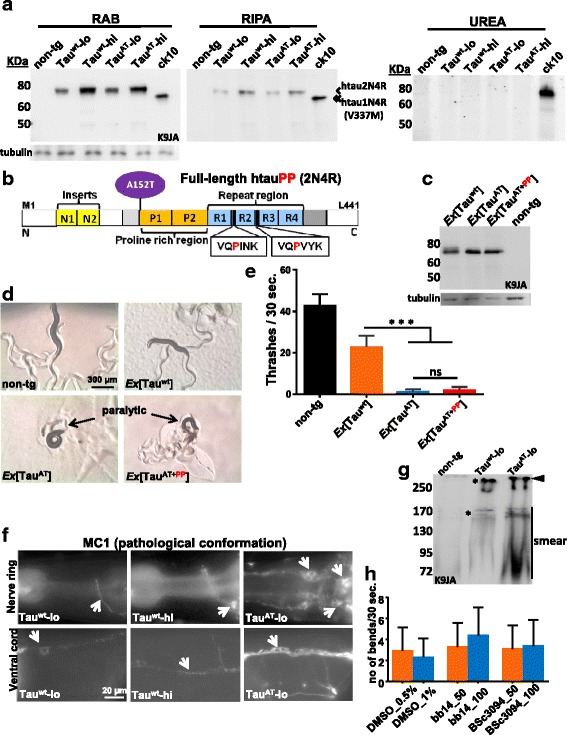
Mutant Tau^AT^ does not aggregate, adopts a pathological conformation and anti-aggregation compounds do not rescue the paralysis. **a** Sequential extraction of tau from aged animals (mixed stage consisting worms of mostly > 3 days old). *CK10* line transgenic for htauV337M (1N4R) accumulates detergent insoluble tau (insoluble aggregated tau); while no insoluble tau is detected in Tau^wt^ or Tau^AT^ lines. 2N4R-tau (single arrow) in Tau^wt^ and Tau^AT^ lines migrates slower than the 1N4R-tau (double arrow) in *CK10* worms. With age, more Tau accumulates in the detergent soluble fraction (representing Tau bound to membranous structures) (see also Additional file 13A for day-1 old animals). **b** Bar diagram of anti-aggregant Tau construct Tau^AT+PP^ with A152T mutation plus two additional proline substitutions in the hexapeptide motifs (htau40A152T*I* ^*277*^ *PI* ^*308*^ *P*). **c** 30 day-1 adult animals from non-tg, *Ex*[Tau^wt^], *Ex*[Tau^AT^] and its anti-aggregant variant *Ex*[Tau^AT+PP^] after lysis in 1 x sample buffer, then subjected to 10 % PAGE and subsequent western blot analysis using K9JA-pan-tau antibody. *Ex*[Tau^wt^], *Ex*[Tau^AT^] and its anti-aggregant variant *Ex*[Tau^AT+PP^] carrying the respective Tau transgenes as extrachromosomal arrays (*Ex*) show comparable levels of Tau expression. Tubulin serves as loading control. **d** Micrographs of the worms. The anti-aggregant variant of mutant Tau^AT^ (Tau^AT+PP^) is equally toxic and produces a similar paralytic phenotype as the single mutant Tau^AT^. Note the absence of tracks and coiled body in *Ex*[Tau^AT+PP^] similar to *Ex*[Tau^AT^]. **e** Mean thrashing assay of day-1 old adult animals carrying Tau^wt^, Tau^AT^ or its anti-aggregant variant (Tau^AT+PP^) transgenes as extrachromosomal arrays. *Ex*[Tau^AT+PP^] shows less thrashes than the non-tg (~5 % of non-tg) or *Ex*[Tau^wt^] worms (~9 % of Tau^wt^ worms). But there is no difference between the *Ex*[Tau^AT^] and its anti-aggregant variant *Ex*[Tau^AT+PP^], indicating that the toxicity does not depend on amyloidogenic aggregation. Non-tg strain serves as control. Error bars denote SEM, n ≥ 30. ***P < 0.001, ns., not significant. One-way ANOVA with Tukey’s test applied for multiple comparisons. **f** Immunostaining of day-1 old Tau^wt^-lo, Tau^wt^-hi and Tau^AT^-lo with conformation-specific antibody MC1. Tau in Tau^AT^-lo worms adopts a pathological state as seen by dense staining in the nerve ring and ventral cord. White arrows show stained neuronal processes either in the nerve ring or ventral cord region. Tau^wt^ (-lo and -hi) show only mild staining occasionally. **g** Worm extracts prepared from mixed stage adults in buffer C, resolved by native PAGE and immunoblotted with K9JA show Tau enriched in soluble high molecular weight complexes in Tau^AT^ worm extracts. In addition to two bands common to both Tau^wt^-lo and Tau^AT^-lo lysates (~170 KDa and >250 KDa marked by asterisks), a smear in the range of 72-95 KDa corresponding to lower oligomeric species and a higher band (> > 250 KDa, black arrowhead) can be seen in the Tau^AT^-lo lysate. **h** Mean number of bends per 30 s of Tau^AT^-lo worms treated either with DMSO (solvent control) or with 50 or 100 μM concentrations each of known aggregation inhibitors of Tau (Rhodanine compound bb14, PTH compound BSc3094 in DMSO). The lack of rescue indicates that the toxicity of Tau^AT^ is based on some mechanism distinct from aggregation (for comparison see [17]). Error bars denote SEM. One-way ANOVA with Tukey’s test applied for multiple comparisons (ns., not significant)

A common feature of neurodegenerative diseases is protein aggregation. There is an ongoing debate regarding the nature of the toxic species, e.g. the relatively stable insoluble aggregates which could obstruct the cellular space, or soluble aggregates in the form of oligomers [38–40]. To address this question for the case of the Tau^AT^ mutant, we first biochemically extracted tau from day-1 old worms [17]. Soluble fractions were completely extracted first by using a salt buffer (RAB), which removes most of the soluble cytoplasmic fraction, followed by a detergent buffer (RIPA), which removes protein-protein or protein-membrane complexes. The detergent insoluble pellet was finally reextracted with urea buffer (Urea). Neither Tau^wt^ nor Tau^AT^ accumulated detergent insoluble tau at day-1 (Additional file 13). Next we extracted tau from mixed stage worms consisting mostly of > 3 days old ones. Here we used *CK10* line which is transgenic for htau^V337M^ (1N4R) and is known to accumulate detergent insoluble tau with age (mixed stage) as a positive control [41]. The *CK10* line but not Tau^wt^ or Tau^AT^ showed tau in the detergent insoluble fraction (Fig. 8a), although there was an increase in the detergent soluble Tau fraction in Tau^wt^ and Tau^AT^ with age compared to the day-1 old adults (see Additional file 13 for comparison).

To confirm our results, we generated another line expressing a Tau chimaera with two additional proline substitutions in the hexapeptide motifs (I277P and I308P), besides the A152T mutation (termed Tau^AT+PP^) (Fig. 8b). The proline substitutions make this Tau chimaera inherently incapable of forming detergent insoluble Tau filaments because they block the amyloidogenic β-structure [42]. We then compared the protein levels in the worms carrying Tau^wt^, Tau^AT^ or Tau^AT+PP^ transgenes as extrachromosomal arrays at day 1, all of which showed comparable levels of Tau protein (Fig. 8c). Contrary to expectations, Tau^AT+PP^ resulted to be equally toxic and the Tau^AT+PP^ worms were as paralytic as single mutant Tau^AT^ worms (Fig. 8d,e). This argues that the toxicity does not depend on the formation of β-structure and incipient aggregation, in contrast to the pro-aggregant mutation ΔK280 described earlier [17, 43].

Before the accumulation of insoluble aggregates as tangles, Tau is known to undergo a change towards a pathological conformation which accompanies oligomerization during the pre-tangle state and can be recognized by antibodies Alz-50 or MC-1 [44]. Using MC-1 staining, we confirmed pathological conformation and observed an extensive staining of nerve ring ganglion and nerve cords in Tau^AT^-lo worms. By contrast, Tau^wt^ (-lo and -hi) worms showed only minimal staining (Fig. 8f) and non-tg worms showed no staining (data not shown). Since it was reported previously that Tau^AT^ mutant tau has a higher tendency to form oligomers in vitro [6], we wanted to test if such oligomeric species also exist in Tau^AT^ worms. To detect oligomers, we applied native PAGE to analyze total lysate extracted from the mixed stage worms in buffer C (see Methods) using K9JA antibody. Lysate from Tau^wt^-lo showed protein accumulated in two bands (~170 KDa and >250 KDa marked by asterisk in Fig. 8g). By contrast, the lysate from Tau^AT^-lo showed in addition a higher band (Fig. 8g, black arrowhead) at the top of the gel and a smear in the range of 72- 95 KDa (Fig. 8g, line), meaning that Tau^AT^ worms accumulate a broad range of higher molecular weight tau species compared to Tau^wt^ worms. Lysate from non-tg worms on the other hand did not show any reaction to K9JA antibody. We conclude that the mutation A152T favors a pathological conformation which might promote oligomerization.

### The toxicity of mutant Tau^AT^ is not prevented by anti-aggregation compounds

The conclusion that the toxicity of A152T mutation occurred independently of the β-propensity of the repeat domain was corroborated by treatment of Tau^AT^-lo worms with inhibitors of Tau aggregation (Rhodanine compound bb14 and PTH compound BSc3094), that were competent to ameliorate the phenotype of a worm model based on tau aggregation [17]. The Tau^AT^-lo worms were treated with varying concentrations of compounds in liquid culture for three days, starting from the L1 larval stage. However, the compounds failed to improve the paralytic phenotype of the Tau^AT^-lo worms (Fig. 8h). This implies that the principle behind the pathological features observed in Tau^AT^ worms is perhaps different from that of the conventional Tau-aggregation.

### The C-terminal half of Tau including the repeat domain is necessary for the toxicity of human tau in *C. elegans* neurons

**Fig. 9:**
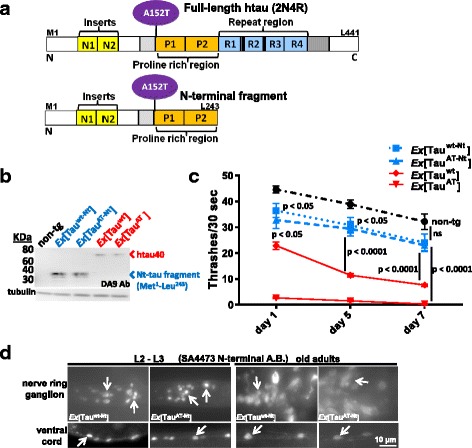
N-terminal tau fragments (wild-type or with A152T mutation) are not toxic to *C. elegans* neurons. **a** Bar diagram depicts the N-terminal fragment of tau (amino acids Met^1^-Leu^243^) derived from full-length Tau. The pan-neuronal *snb-1* promoter drives the expression of wild-type N-t- or mutant N-t-fragment (Tau^wt-Nt^ and Tau^AT-Nt^ respectively) in *C. elegans* neurons. **b** Blot showing the protein expression levels in worms carrying N-terminal fragments or full-length tau transgenes as extrachromosomal arrays. 30 synchronized day-1 old adult worms from each transgene (*Ex*[Tau^wt-Nt^], *Ex*[Tau^AT-Nt^], *Ex*[Tau^wt^] and *Ex*[Tau^AT^]) were lysed and subjected to western blot analysis using N-terminal Tau specific DA9 antibody (epitope at aa 100-130). Non-tg worms serve as control and tubulin as loading control. **c** Age-related comparison of rate of body thrashing in liquid for days 1, 5 and 7 from the respective transgenic animals. In contrast to full-length tau (Tau^wt^ or Tau^AT^), expression of N-terminal Tau fragments in *C. elegans* neurons (Tau^wt-Nt^ or Tau^AT-Nt^) cause only slight reductions (~10-13 %) in thrashing at day 1 and day 5. The data points represent the mean (±SEM) thrashing rate and time point, n ≥ 30. Two-way ANOVA followed by Bonferroni correction was used for multiple comparisons. **d** Immunostaining of animals expressing either wild-type- or mutant-N-t tau fragments at different time points with N-terminal tau-specific antibody SA4473. Top panels depict nerve ring ganglion while the bottom panels show nerve cords. N-terminal Tau fragments show localization restricted to cell bodies and nuclei (white arrows), as nerve processes are largely invisible, in contrast to the situation with full-length Tau (see Fig. 1c)

The repeat domain of tau in the C-terminal domain harbors the primary physiological and pathological functions of tau, stabilization of microtubules and formation of aggregates in disease [45, 46]. Much less is known about the functions of the N-terminal domain; but it is known to interact with motor proteins and signalling proteins [9, 47–49]. Additionally, the pathological conformation adopted by tau is achieved as a result of intramolecular folding of C-terminal against the N-terminal domains (discontiguous epitope MC-1, residues 5-15 + 312-322) [44]. Furthermore, mimicking the post-translational modifications at epitopes of antibodies AT8, AT100 and PHF-1 (upstream and downstream of the repeats, resp.) leads to a change of the paperclip folding of Tau that is known to consolidate the MC-1 epitope [50]. Since the A152T mutation lies in the N-terminal projection domain of tau, we wondered if N-terminal tau domains that lack a potential MC-1 epitope would still produce a similar phenotype as the full-length tau. We generated transgenic worms expressing pan-neuronally the N-terminal tau (Nt-tau) chimaeras (Met^1^-Leu^243^) with or without the mutation A152T (Fig. 9a). Several lines carrying the N-terminal tau transgenes with or without the mutation (*Ex*[Tau^AT-Nt^] and *Ex*[Tau^wt-Nt^] respectively) as extrachromosomal arrays were obtained by expressing the respective transgenes at comparable levels. However, compared to worms expressing full-length htau40 (Tau^wt^ or Tau^AT^) as extrachromosomal arrays (*Ex*[Tau^wt^] and *Ex*[Tau^AT^] respectively), worms expressing N-terminal fragments (*Ex*[Tau^wt-Nt^] and *Ex*[Tau^AT-Nt^]) showed much higher expression (5-10 fold) (Fig. 9b). In spite of these high levels, the N-terminal tau fragments produced only a slight reduction in mobility (thrashes) compared to non-tg at day1 and day 5, and the difference became statistically insignificant at day 7 (Fig. 9c). These results are consistent with earlier findings where an N-terminal fragment of Tau expressed in mouse brain produced phenotypically normal mice [48].

We then performed staining of worms expressing the N-terminal tau fragments with the polyclonal antibody SA4473 specific for the N-terminal half of tau. Nt-tau fragments in these worms showed extensive staining exclusively in somata and nuclei, but were largely excluded from the neuronal processes (Fig. 9d). These results show that N-terminal tau fragments which are unable to move into the neuronal processes are not toxic to *C. elegans* neurons. Thus a mild uncoordinated phenotype due to Tau^wt^ and a strong paralytic phenotype due to Tau^AT^ could both be abolished by preventing the Tau chimaeras to move into the neuronal processes. In conclusion, a severe toxicity occurs only when both conditions are met; (a) presence in neuronal processes, (b) A152T mutation. Therefore, we speculate that it might be the ability of full-length tau to move into different neuronal processes owing to its C-terminal half that makes it toxic and the A152T mutation further aggravates the problem, whereas restricting its localization to the cell body by abolishing the C-terminal half renders it non-toxic, even in the mutant form. Table 3 sums up the data on the effect of various Tau variants when expressed pan-neuronally in *C. elegans*.

**Table 3:** Summary of different Tau variant transformations in *C. elegans* neurons (Toxicity index: %age reduction in thrashes when normalized to non-tg)

Strain	Sub-cellular localization	Phenotype (Unc)	Toxicity index
non-tg		Non	0
Full-length (2N4R) hTau40^wt^-lo	Cell bodies and neuronal processes excluding nucleus	Weak	~30
Full-length (2N4R) hTau40^wt^-hi	Cell bodies and neuronal processes excluding nucleus	Mild	~60
Full-length (2N4R) hTau40^AT^ (lo/hi)	Cell bodies and neuronal processes excluding nucleus	Strong	~90
Full-length (2N4R) hTau40^AT+PP^	Cell bodies and neuronal processes excluding nucleus	Strong	~90
N-terminal hTau^wt^ (Met^1^-Leu^243^)	Cell bodies and neucleus excluding neuronal processes	No obvious phenotype	~13
N-terminal hTau^AT^ (Met^1^-Leu^243^)	Cell bodies and neucleus excluding neuronal processes	No obvious phenotype	~15

Transgenic lines based on different Tau constructs are compared to the non-transgenic control (N2) animals

## Discussion

In this study we used a *C.elegans* model to examine the toxicity of a rare mutation in the MAPT gene (A152T*MAPT*) that was recently discovered as a risk factor for frontotemporal dementia spectrum disorders (FTD, PSP, CBD, AD, and others) [51]. Considering that most Tau mutations lie in or near the repeat domain and thus affect Tau-microtubule binding and Tau aggregation, the A152T mutation is unusual in that it lies in the proline-rich region far upstream of the repeat domain [7, 8], with only weak effects on Tau aggregation [6]. This poses the question of an alternative mechanism of toxicity. Over the years, several functions have been assigned to the N-terminal domain of tau which do not coincide with the functions of the repeat domain [9]. Examples of interactors with the N-terminal domain are p150/Dynactin [47, 52], or signalling kinases containing SH3 domains [48, 49, 53], which point to functions distinct from microtubule stabilization.

We found that elevation of wild-type human Tau (Tau^wt^) in *C. elegans* neurons is already mildly toxic and can induce late-onset pathology in a dose-dependent manner, consistent with results from mouse models of AD [54]. However, mutant Tau^AT^ at similar levels induces a much more pronounced pathology with an early onset. These worms developed severe motor dysfunction, paralysis and neurodegeneration of GABAergic neuronal processes (Figs. 1 and 2). Since we observed neurodegeneration in motor neurons, a condition often associated with old age in humans [55], we hypothesized that Tau^AT^ could cause effects similar to those observed in aging neurons. This can be tested on mechanosensory neurons by well-defined parameters [24], and indeed the neurons of Tau^AT^ worms showed early signs of morphological abnormalities such as bendings and extra outgrowths from neuronal processes and somata (Fig. 3b, c) compared to non-tg and Tau^wt^ worms. The severity of these morphological abnormalities increased with age (Fig. 3c). Moreover, these signs of early aging on the level of Tau^AT^-expressing neurons corresponded to a drastic reduction in life-span of the animals as well (Fig. 3e). Since mutations in genes that reduce the life-span also cause early onset of age-dependent morphological abnormalities in neurons [23, 24, 56], it is safe to speculate that the pathways that are involved in inducing such age-dependent morphological abnormalities might get activated early in Tau^AT^ worms. However, some alternate pathways that induce similar morphological changes in neurons independently of aging have also been reported [23, 34].

What could be the reasons behind the neuronal dysfunction and age-related phenotypes in Tau^AT^ worms? Tau interacts with microtubules, microtubules are the tracks for intracellular transport, and defects in neuronal transport machinery have been linked to neurodegeneration. This led us to search for signs of transport defects in the neurons of Tau^AT^ worms. This was done for the cases of two major neuronal cargoes, namely synaptic vesicle (carrying Rab-3 GTPase) and mitochondria [57]. The Tau^AT^ worms presented a perturbed distribution of both of these cargoes. For example, mCherry::RAB-3 puncta accumulated in distal axons and in cell bodies but were depleted in the mid region of mechanosensory neurons (Fig. 4, Additional file 12), consistent with defective retrograde and anterograde transport [58–60]. Moreover, not only the transport but the integrity of the neuronal compartments was compromised in mutant Tau^AT^ worms as suggested by the mislocalization of mCherry::RAB-3 in the dendritic compartments of polarized PVD neurons (Fig. 5b, c) [33, 35].

Polarized sorting of synaptic vesicles requires ankyrin (UNC-44) driven anchoring of CRMP (UNC-33) into axons, which in turn drives plus-end directed microtubules extending into the axons and subsequent plus-end directed axonal traffic by kinesin (UNC-104) [35]. The observed missorting of synaptic vesicle associated RAB-3 into dendrites of Tau^AT^ worms could be caused by several factors, e.g. more plus-end directed microtubules in dendrites, or mislocalization of ankyrin (UNC-44) and subsequent localization of CRMP (UNC-33) into dendrites, both of which are normally excluded from this compartment. The resulting changes in the dendritic microtubular network due to CRMP could then trigger kinesin (UNC-104) dependent traffic into the dendrites. Future studies should reveal if this phenomenon in Tau^AT^ worms is related to a direct interaction of tau with ankyrin (UNC-44) or CRMP (UNC-33). In addition to the microtubular network, tau is also known to interact and directly or indirectly influence the dynamics of the actin cytoskeleton [61], and this cross-talk with the microtubule- and actin-cytoskeleton might play a critical role in tau-induced neurodegeneration [62]. In line with these findings, two suppressors (*sut-1* and *sut-2*) have been identified in a genetic screen using *C. elegans* [15, 16]. Although a direct interaction between tau and SUT-1 or SUT-2 is not known; however, both of these proteins interact with proteins associated with either microtubule-based or actin-based cytoskeletal systems.

A similar correlation was seen with mitochondrial particles tagged with YFP in DA9 motor neurons. Mutant Tau^AT^ worms showed less mitochondrial particles in the synaptic areas of DA9 axons. Time-resolved imaging revealed that this perturbed mitochondrial distribution is due to a decreased mitochondrial trafficking in Tau^AT^ worms (Figs. 6 and 7). This perturbed distribution of neuronal organelles is analogous to the previous *C. elegans* kinesin/dynein double mutant studies which reported an overall decrease in the number density of synaptic vesicles along the axonal processes and accumulation of very few vesicles in the end neurons. This can be explained by the fact that both retrograde and anterograde transport processes are affected in a kinesin/dynein double mutant. On the other hand, the expression of Tau^wt^ generated a phenotype analogous to a dynein mutant where a deficient retrograde transport leads to the accumulation of synaptic vesicles along the distal part of the axon [59, 60]. These transport deficits might also explain the extensive neuronal restructuring of the touch neurons in Tau^AT^ animals (Fig. 3). The fact that most of these non-specific branches grew directly from the cell bodies supports the view that mitochondria accumulate in the cell bodies which may provide local energy for new outgrowths. Occasionally these non-specific branches in touch neurons of Tau^AT^ worms underwent further branching, consistent with earlier studies that attributed this phenomenon to ROS production by static dysfunctional mitochondria [24]. Overtime, even wild-type Tau^wt^ worms started showing these phenotypes, albeit to a lesser extent.

It is worth noting that in certain assays, Tau^wt^ and Tau^AT^ worms did not differ much, possibly because a threshold in these phenotypic assays is lower and even the wild-type tau was able to cross that threshold, albeit at higher levels. For instance, Tau^wt^ at higher levels led to pre-synaptic defects similar to Tau^AT^ at both lower and higher levels (Fig. 5d, e). Similarly, there was no change in the phosphorylation status of tau in both Tau^wt^ and Tau^AT^ worms (Additional file 14). This indicates that there is no special preference for mutant tau with regard to phosphorylation by *C. elegans* kinases and further suggests that tau phosphorylation may not be a cause for toxicity. In addition, neither Tau^wt^ nor Tau^AT^ worms accumulated insoluble tau (Fig. 8a).

Aggregation is usually considered a hallmark of Tauopathies such as AD, PSP, or CBD, but there is also persuasive evidence that the toxic species are not the fully developed insoluble and aggregated forms of the protein but earlier soluble oligomeric precursors [63–65]. We therefore searched for assembly forms of Tau^AT^ in the transgenic worms. The results showed that there are no detectable insoluble aggregates (as judged by the usual standard isolation procedures), but an increase in soluble oligomeric forms. In this regard, the Tau^AT^ are distinct from other cases where aggregation-prone Tau mutants were expressed and accumulated in the insoluble fraction in *C. elegans* neurons, e.g. mutants V337M or ΔK280 [17, 41]. The absence of insoluble aggregates was further confirmed in two ways: (i) by expressing an inherently aggregation-incompetent Tau construct with two proline substitutions in the hexapeptide motifs plus the A152T mutation which yielded a similar phenotype as a tau construct with the A152T mutation alone (Fig. 8 d, e); and (ii) by treating the Tau^AT^ worms with known aggregation inhibitor compounds, which offered no relief (Fig. 8h), in contrast to worms expressing pro-aggregant forms of Tau [17]. These experiments argue that the toxic principle of Tau^AT^ does not depend on aggregation. Important to mention here is that another line expressing a full-length htau40A152T chimaera with an additional V337M mutation (*Ex*[Tau^AT+VM^]) also gave a similar phenotype as the *Ex*[Tau^AT^] line expressing the single mutant tau chimaera htau40A152T (Additional file 13C,D). Keeping in mind that the A152T mutation lies in a region important for scaffolding and signalling pathways, it further suggests that the mutation leads to a toxic gain of function distinct from aberrant aggregation. Indeed, the Tau^AT^ worms showed strong staining with antibody MC-1 (Fig. 8f), a characteristic feature of pathological conformational changes [44]. Thus the situation is reminiscent of other neurodegenerative conditions where soluble pre-aggregates are the primary culprits behind the toxicity [66–68]. In line with these findings, tau in A152T worms was detected mostly enriched in the soluble higher molecular weight species (potential oligomers) by native PAGE (Fig. 8g). A growing body of evidence suggests that a cell combats the decline of proteostasis due to toxic gain of function of proteins by sequestering soluble proteins into insoluble aggregates [69, 70]. The fact that Tau^AT^ worms have a short life-span (Fig. 3e) might explain that there is not enough time to sequester and circumvent toxic effects caused by soluble diffusible tau. It would be of interest to investigate whether an increase of life-span, e.g. by suppressing insulin-signalling [71], would allow them to drive these soluble tau species into insoluble aggregates, and subsequently whether the pathological consequences are alleviated.

One important finding of this study was that the Tau^AT^ mutant does not induce pathology unless tau moves into the neuronal processes. It appears that *C. elegans* neurons can easily cope with a tau chimaera, irrespective of whether it is wild-type or a A152T mutant, provided that its localization is restricted to the cell body. The evidence is that when expressing wild-type or A152T mutant N-terminal tau fragments without the repeat domain, their localization was restricted to cell body (Fig. 9d). The explanation is that the fragments cannot engage to the microtubule-based transport machinery since they lack the MT-binding domain [72]. Both these lines were almost as healthy as the non-tg lines (Fig. 9c), even though the N-terminal fragments were expressed at a 5–10 fold higher level than full-length tau (Fig. 9b). Secondly, a full-length variant of Tau with two additional proline substitutions in the hexapeptide motifs (Tau^AT+PP^) which is incompetent for aggregation, but otherwise is transported normally into cell processes (Additional file 15), induces pathology to the same extent as that of Tau^AT^ (Fig. 8e). This supports earlier findings in mice showing that tau needs to move out of the cell body in order to exert its toxic effects [48]. In the light of the current interest in the cell-to-cell spreading of Tau it would be interesting to know whether the intercellular propagation of Tau^AT^ could contribute to the toxicity. However, this issue cannot be addressed in the current model, as mutant Tau is expressed pan-neuronally.

Taken together, the detailed description of the pathology due to the Tau^AT^ mutation provided by the new worm models opens up multiple possibilities to identify cellular components involved in the Tau-dependent pathology and might contribute to the development of novel therapeutic interventions of tauopathies.

## Conclusions

In this study, we present a new *C. elegans* model to understand the pathological consequences of A152T*MAPT* mutation, recently identified in patients diagnosed with frontotemporal spectrum disorders, including PSP, FTD, CBD, and AD. Using this model we found that, while the wild-type human tau (Tau^wt^, 2N4R) overexpression in *C. elegans* neurons induces a progressive mild locomotor defects in a dose-dependent manner, mutant tau (Tau^A152T^, 2N4R) induces a severe paralysis accompanied by acute neuronal dysfunction. Tau^A152T^ worms show morphological alterations in neurons similar to what has been reported in aging neurons and exhibit a reduced life-span. Tau^A152T^ overexpressing neurons show mislocalization of cellular organelles and disrupted mitochondrial trafficking. Importantly, Tau^A152T^ remains in a pathological conformation but does not accumulate as insoluble aggregates. Moreover, the C-terminal domain of tau which engages with the microtubules is necessary to induce pathology despite the fact that the A152T mutation lies in the N-terminal domain. Together these data show that the mutant Tau^A152T^ overexpression in *C. elegans* neurons induces pathology that is not dependent on aggregation.

## Methods

### Plasmid constructs

To generate A152T cDNA [6], we used site-directed mutagenesis (Stratagene QuikChange reagents) to introduce the point mutation A152T into htau40WT cDNA. The respective cDNAs encoding wild-type htau40WT and mutant htau40A152T were ligated into SalI and KpnI sites of pPD49.26 vector between the *snb-1* promoter and *unc-54* 3′-UTR (a generous gift from Dr. B.C. Kraemer, University of Washington, Seattle). Two additional proline substitutions were introduced into mutant htau40A152T in the hexapeptide motifs (I277P and I308P, intended to break beta-structure) [73]. The resulting cDNA was cloned into SalI and KpnI sites of pPD49.26 as described above, yielding *Psnb-1::htau40A152T(I*^*277*^*P)(I*^*308*^*P)*. Similarly, another Tau chimaera with A152T plus V337M mutation was generated as described above and cloned into SaII and KpnI sites of pPD49.26, yielding *Psnb-1::htau40A152TV337M*. As the A152T mutation lies outside the repeat region of Tau in the N-terminal domain, we also generated worms expressing only the N-terminal regions of tau, residues 1-243. We cloned the first 243 amino acid region of wild-type and mutant tau cDNA into the BamHI and KpnI sites of pPD49.26 to generate the transgene constructs *Psnb-1::N′t-htauWT*^*Met1-Leu243*^ and *Psnb-1::N′t-htauA152T*^*Met1-Leu243*^ respectively. All plasmid constructs were confirmed by DNA sequencing.

### Generation of transgenic lines

Complex arrays were generated by injection of Tau transgenes along with a coinjection marker (*Pmyo-2::gfp* or *Pmyo-2::mCherry,* gift of Dr. R. Baumeister) into the gonad of N2 (Bristol) at 75 ng/μl. Coinjection markers were used at a concentration of 20 ng/μl and also served as controls when injected alone at the same concentrations. To generate the stable lines, the transgenes were integrated by exposing the animals to 300 J/m^2^ UV dose [74]. Multiple stable transgenic lines were isolated, and before proceeding further, the transgenic lines were backcrossed to N2 wild-type at least five times to eliminate any background mutations.

*C. elegans* maintenance and crosses were performed according to the standard methods [75]. Worms were cultured at 20 °C unless otherwise specified. N2 Bristol was used as the wild-type *C. elegans*. The following transgenic strains were used: PIR1: *pirEx1*[*Psnb-1::htau40WT;Pmyo-2::gfp*], PIR2: *pirEx2*[*Psnb-1::htau40A152T;Pmyo-2::gfp*], PIR3: *pirIs3*[*Psnb-1::htau40WT-low;Pmyo-2::gfp*], PIR4: *pirIs4*[*Psnb-1::htau40WT-high;Pmyo-2::gfp*], PIR5: *pirIs5*[*Psnb-1::htau40A152T-low;Pmyo-2::gfp*], PIR6: *pirIs6*[*Psnb-1::htau40A152T-high;Pmyo-2::gfp*], PIR7: *pirEx7*[*Psnb-1::htau40A152T(I*^*277*^*P)(I*^*308*^*P);Pmyo-2::mCherry*], PIR8: *pirEx8*[*Psnb-1::N′t-tauWT*^*Met1-Leu243*^*;Pmyo-2::mCherry*], PIR9: *pirEx9*[*Psnb-1::N′t-tauA152T*^*Met 1-Leu243*^*;Pmyo-2::mCherry*], CZ1197: *juIs73*[*Punc-25::gfp*]III (gift of Dr. E. Lundquist), PIR10: *pirIs3;juIs73*, PIR11: *pirIs4;juIs73*, PIR12: *pirIs5;juIs73*, PIR13: *pirIs6;juIs73*, *zdIs5:*[*Pmec-4::GFP + lin-15(+)*]*,* PIR14*: pirIs3;zdIs5,* PIR15*: pirIs4;zdIs5,* PIR16*: pirIs5;zdIs5*, *vdEx262:*[*Pmec-4::mCherry::rab-3;Punc122::gfp*] (gift of Dr. M. Hilliard), PIR17*: pirIs3;vdEx262,* PIR18*: pirIs4;vdEx262,* PIR19*: pirIs5;vdEx262, wyEx2709:* [*Pitr-1::TOM-201-54aa::yfp;Podr-1::gfp*] (gift of Dr. K. Shen), PIR20: *pirIs3;wyEx2709*, PIR21: *pirIs4;wyEx2709*, PIR22: *pirIs5;wyEx2709*, *jsIs609:Is:*[*Pmec-4::MLS::gfp*] (gift of Dr. Nonet M), PIR23: *pirIs3; jsIs609*, PIR24: *pirIs4; jsIs609*, PIR25: *pirIs5;jsIs609*, *kyIs445:Is:*[*Pdes-2::mCherry::RAB-3;des-2::SAD-1::GFP;odr-1::DsRED*] (gift of Dr. C.I. Bargmann), PIR26: *pirIs3;kyIs445,* PIR27: *pirIs4;kyIs445,* PIR28: *pirIs5;kyIs445*, PIR29: *pirEx29*[*Psnb-1::htau40A152TV337M;Pmyo-2::mCherry*], CK10:*bkIs10*[*Paex-3::htauV337M(1N4R);Pmyo-2::gfp*], CB211: *lev-1(e211)*IV and NM791: *rab-3(js49)*II.

### Behavioral assays

For qualitative locomotor behavior, synchronized day-1 old adults were placed onto the center of NGM plates freshly spotted with *E. coli* OP-50 and photographed after 10 min. For thrash assays, synchronized animals from each transgenic line were transferred into 20 μl of M9 buffer (22 mM KH_2_PO_4_, 42 mM Na_2_HPO_4_, 86 mM NaCl and 1 mM MgSO_4_) on a glass slide. After allowing the animals to settle for 1 min, the frequency of body bending was counted for 30 s [76].

### Life span assay

A synchronous population of worms was obtained by extracting eggs from gravid adults and incubated in M9 buffer overnight. Synchronised L1 larvae were collected and allowed to grow until late L4 stage on regular NGM plates seeded with *E. coli* OP50, and then transferred onto OP50 seeded NGM plates containing 0.05 mg/ml FUDR (5-fluoro-2-deoxyuridine) for the first two days to inhibit growth of progeny. Thereafter, the worms were grown again on regular OP50 seeded NGM plates and scored every two days by tugging gently on the tail with a platinum wire covered with bacteria. Worms that failed to respond to touch were scored as dead. GraphPad Prism software was used to perform the statistical analysis.

### Immunohistochemistry

Worms were permeabilized by freeze cracking after fixation in 2 % paraformaldehyde solution as described previously [58]. Polyclonal rabbit pan-tau antibody (K9JA; Dako A-0024) at a dilution of 1:5000 was used to detect Tau. To monitor the conformational changes, we used the pathological conformation-specific monoclonal mouse antibody MC-1 [77]; (gift from Dr. P. Davies; 1:20). For immunostaining of worms expressing N-terminal tau fragments, rabbit polyclonal antibody SA4473 (Eurogentec; 1:300) was used. Respective secondary antibodies were used at 1:350 dilutions. Stained animals were imaged at 63x using a Zeiss inverted epifluorescence microscope.

### Imaging

GABAergic motor neurons were imaged by mounting immobilized young adult animals in 50 mM sodium azide (Sigma) on glass slides with 2 % agarose pads, and imaged using a 20x or 40x objective on LSM700 (Zeiss). To study the steady state distribution of synaptic vesicles, worms were anesthetized in 50 mM sodium azide, mounted on 2 % agarose pads, and neurons were imaged at 63x with Zeiss inverted epifluorescence microscope. For steady state imaging of mitochondrial marker *Pitr-1:TOM-20*^*1-54aa*^*::yfp*, we used Nikon microscope equipped with Andor Spinning Disc Setup and EM-CCD camera (Andor iXon 3). For live imaging of GFP tagged mitochondria, worms of appropriate age (day-1 and day-3 old adult) were anesthetized in 2-3 mM of levamisole (Biomol) [58] and mounted on 2 % agarose pads. Time lapse (3fps) images of mitochondria tagged with GFP in mechanosensory neurons were acquired at 63x with a Zeiss epifluorescence microscope equipped with a CCD (Photometrics) camera. Images were acquired for constant time (t = 2.5 min) in two different regions of a neuron, in the proximal part (axonal part adjacent to cell body, ~60–80 μm) and middle part ~120 μm away from cell body (refer to schematic in Fig. 4a). The number of moving mitochondrial events was counted manually for each genotype from the acquired movies. Representative kymographs were generated using an ImageJ (NIH) plug in.

### Aldicarb and levamisole assays

To perform the aldicarb (acetylcholine esterase inhibitor) and levamisole (acetylcholine receptor agonist) sensitivity assays, day-1 old adult worms were transferred to plates containing 1 mM aldicarb and 0.2 mM levamisole, respectively and the time course of paralysis was assayed as described previously [36, 78]. 20 animals per strain were scored for paralysis and each experiment was repeated three times.

### Pharmacological treatment

Chemical treatment was applied in liquid cultures as described earlier [17]. Aggregation inhibitor compounds bb14 (Rhodanine) or BSc3094 (PTH) were dissolved in DMSO and added to the liquid culture at 50 and 100 μM concentrations.

### Protein extraction

To extract total Tau protein, synchronized worms were washed off NGM plates with water and the bacteria were removed in the subsequent washing steps. The resulting worm pellets (~100 mg) were resuspended in 1X protein sample buffer containing 355 mM 2-mercaptoethanol and boiled at 96 °C for 10 min. with continuous shaking at 14,000 rpm. To determine the accumulation of insoluble tau aggregates, a slight modification of the previous extraction protocol [17] was performed using buffers of increasing stringency. Briefly, after removing the dead animals and bacteria by flotation on a 30 % sucrose solution, worm pellets (~100 mg) were resuspended in twice the amount (w/v) of high-salt RAB buffer [100 mM 2-(N-morpholino) ethanesulfonic acid (MES), 1 mM EGTA, 0.5 mM MgSO_4_, 20 mM NaF]. Worm pellets were lysed completely by sonication (6 × 10 s, 10 s break) on ice, and homogenates centrifuged at 40,000 g for 40 min. The supernatant is the soluble RAB fraction. RAB pellet was sonicated for 10 s in 1 M sucrose containing RAB buffer and centrifuged for 20 min at 40,000 g, and the supernatant was discarded. The pellet was extracted with RIPA buffer (150 mM NaCl, 1 % Nonidet P-40, 0.5 % deoxycholate, 0.1 % SDS, 50 mM Tris, pH 8.0) and centrifuged at 40,000 g for 20 min. The supernatant constitutes the detergent soluble RIPA fraction. The resulting pellet, after a brief washing with RIPA buffer, was extracted with urea containing buffer (UREA) [30 mM Tris, 7 M urea, 2 M thiourea, 4 % CHAPS (3-[(3-cholamidopropyl)dimethylammonio]-1-propanesulfonate), pH 8.5] and centrifuged at 13,000 g for 15 min. The supernatant is the detergent insoluble fraction.

For native PAGE, frozen worm pellets (~100 mg) were resuspended in buffer C (20 mM Hepes, pH 7.9, 25 % glycerol, 0.42 M NaCl, 1.5 mM MgCl_2_, 0.2 mM EDTA, 0.5 mM DTT) and lysed by sonication (2 × 10 s, 10 s break) on ice. After a brief centrifugation at 40,000 g for 5 min, the supernatants were analyzed on 4–12 % native PAGE. The entire extraction procedures were carried out on ice and centrifugation steps were at 4 °C. All buffers contained Complete Protease Inhibitor Mixture 3× (Sigma-Aldrich P8340, Hamburg, Germany), 1 μM okadaic acid (phosphatase inhibitor) and 0.5 mM PMSF (protease inhibitor).

## Supplementary methods

### Immunoblotting

Worm fractions (total or sequentially extracted - salt soluble, detergent soluble or detergent insoluble) were migrated in 10 % polyacrylamide gels, transferred to nitrocellulose membranes (Immobilon) and immunoblotted. The following antibodies were used: DM1α-tubulin (1:500; Sigma), K9JA (1:20,000; no. A0024; Dako), AT8 (1:500; Thermo Scientific), PHF-1 (1:500; a gift from Dr. P. Davies, Albert Einstein College, Bronx, USA), 12E8 (1:500; Dr. P. Seubert, Elan Pharmaceuticals, South San Francisco, USA), AT180 (1:500; Thermo Scientific), AT100 (1:500; Thermo Scientific). For worms expressing the N-terminal region of tau, antibody DA9 (raised against aa100-130, a gift from Dr. P. Davies) was used at a dilution of 1:250. Peroxide-conjugated secondary antibodies and ECL solution (Thermo Scientific) were used to visualize the blots. AIDA software (Raytest, Germany) was used to perform densitometry.

## Additional files

Additional file 1: Movie S1. Thrash assay performed with day-1 old Tau^wt^-lo in M9 buffer. (MOV 3770 kb) Additional file 2: Movie S2. Thrash assay performed with day-1 old Tau^wt^-hi in M9 buffer. (MOV 6133 kb) Additional file 3: Movie S3. Thrash assay performed with day-1 old Tau^AT^-lo in M9 buffer. (MOV 3920 kb) Additional file 4: Movie S4. Thrash assay performed with day-1 old Tau^AT^-hi in M9 buffer. (MOV 3326 kb) Additional file 5: Movie S5. Representative time lapse imaging showing the movement of MLS::GFP labelled mitochondria in touch neuron of a non-tg. The cell bodies in all the movies (not visible) are to the left. White arrow marks anterogradely and pink retrogradely moving mitochondria. Movies are displayed at 10x speed, scale bar: 5 μm. (MOV 432 kb) Additional file 6: Movie S6. Representative time lapse imaging showing the movement of MLS::GFP labelled mitochondria in touch neuron of a Tau^wt^-lo. (MOV 200 kb) Additional file 7: Movie S7. Representative time lapse imaging showing the movement of MLS::GFP labelled mitochondria in touch neuron of a Tau^wt^-hi. (MOV 311 kb) Additional file 8: Movie S8. Representative time lapse imaging showing the movement of MLS::GFP labelled mitochondria in touch neuron of a Tau^AT^-lo. (MOV 255 kb) Additional file 9: Pan-neuronal expression of human tau in *C. elegans* by synaptobrevin promoter. Immunochemistry of Tau^wt^-hi and its mutant counterpart Tau^AT^-hi with pan-tau antibody K9JA showing Tau staining in the nervous system. Panels on the left depict nerve ring ganglion, panels on the right side show the ventral cord region. K9JA pan-tau antibody does not stain wild-type non-tg worms (data not shown). (PDF 110 kb) Additional file 10: Mutant Tau^AT^ transgene present as extrachromosomal arrays induces severe locomotion defects in *C. elegans*. (A) Worms carrying human tau-transgene arrays with A152T mutation *Ex*[Tau^AT^] show a severe motor impairment compared to the worms carrying wild-type human tau-transgene arrays *Ex*[Tau^wt^]. Worms were allowed to crawl for 10 min after placing them onto freshly spotted NGM plates before photographing them. *Ex*[Tau^AT^] worms are highly uncoordinated and show a severe paralytic phenotype obvious by the absence of tracks in the *E. coli* lawn and coiling behaviour. Arrows point to the displacement from the origin. (B) 30 day-1 adult animals from non-tg, *Ex*[Tau^wt^] and *Ex*[Tau^AT^] lines were lysed and the lysate was subjected to 10 % PAGE and subsequent western blot analysis using K9JA-pan-tau antibody. Both *Ex*[Tau^wt^] and *Ex*[Tau^AT^] carrying tau transgenes as extrachromosomal arrays show comparable expression of human tau. (C) Mean thrashing rate of day-1 old animals from strains carrying Tau^wt^ and Tau^AT^ transgenes as extrachromosomal arrays. Non-tg wild-type strain serves as control. Error bars denote SEM, n ≥ 30. **P <* 0.05, ***P <* 0.01, ****P <* 0.001. One-way ANOVA with Tukey’s test was applied for multiple comparisons. (D) *Punc-25::gfp reporter* staining GABAergic motor neurons with GFP in non-tg and Tau-transgenic worms. Non-tg control worms show intact ventral and dorsal nerve cords. Transgenic worms carrying Tau-transgenes as extrachromosomal arrays show abnormal GABAergic motor neurons. Worms expressing mutant htau40A152T (*Ex*[Tau^AT^]) show a severe loss of GABAergic neuronal system compared to the worms expressing wild-type tau (*Ex*[Tau^wt^]). Note the gaps (arrow heads) and absence of dorsal cord stretches (bracketed region) in *Ex*[Tau^AT^] worms. (PDF 683 kb) Additional file 11: Expression of mutant Tau^AT^ produces a substantial damage in GABAergic motor neurons. Representative whole worm maximum intensity projections (MIP) of the enlarged insets presented in main Fig. 2. *Punc-25::gfp* reporter that labels GABAergic inhibitory neurons with GFP, was crossed with respective tau-transgenic worms to visualize these neurons. GABAergic neurons show normal connectivity in non-tg worms, both dorsal and ventral nerve cords are intact (A). Expression of Tau^wt^ produced dose dependent abnormalities in GABAergic neurons, with Tau^wt^ -hi neurons (C) accumulating more damage than Tau^wt^ -lo (B). However, mutant Tau^AT^ expression led to severe abnormalities in the form of gaps (arrowheads) in the dorsal and ventral nerve cords, in both Tau^AT^-lo (D) and Tau^AT^-hi (E). Also see Table 1 for detailed quantitative analysis. (PDF 276 kb) Additional file 12: Aberrant localization of presynaptic components in mechanosensory neurons worsens with age in mutant Tau^AT^ worms. (A) Schematic representation of presynaptic cargo distribution in a normal healthy mechanosensory neuron and in an aging neuron. (B) Presynaptic cargo visualized in day-3 old worms after crossing them into *vdEx262:*[*Pmec-4::mCherry::rab-3*] transgene, that expresses mCherry fused to synaptic vesicle associated RAB-3 in mechanosensory neurons. Mislocalization worsens in Tau^AT^-lo, whereas Tau^wt^-lo and Tau^wt^-hi start accumulating mCherry::RAB-3 puncta in the distal axon (yellow arrowheads) and posterior neurite (white arrow). At day 3, the mid neuron of A152T worms becomes almost devoid of mCherry::RAB-3 puncta. (PDF 186 kb) Additional file 13: Mutant Tau^AT^ does not aggregate in *C. elegans* neurons. (A) Sequential extraction reveals no insoluble tau in day-1 old adults. Tau was sequentially extracted from day-1 old transgenic worms, wild-type N2 (Bristol) worms served as control. Worms were lysed by sonication in high-salt buffer (RAB), centrifuged and supernatant collected as salt soluble Tau fraction. Reextraction of salt insoluble pellet with detergent buffer (RIPA) yielded detergent soluble Tau fraction. RIPA insoluble pellet was finally dissolved in urea buffer (UREA) to extract any insoluble fraction. Equal amount of protein was loaded and blotted using K9JA pan-tau antibody. (B) Bar diagram of Tau construct Tau^AT+VM^ with A152T mutation plus additional methionine substitution in the repeat domain (V337M) (htau40A152TV337M). (C) 30 day-1 adult animals from non-tg, *Ex*[Tau^AT^] and the double mutant variant *Ex*[Tau^AT+VM^] after lysis in 1 x sample buffer, then subjected to 10 % PAGE and subsequent western blot analysis using K9JA-pan-tau antibody. *Ex*[Tau^AT^] and *Ex*[Tau^AT+VM^] carrying the respective Tau transgenes as extrachromosomal arrays (*Ex*) show comparable levels of Tau expression. Tubulin serves as loading control. (D) Mean thrashing assay of day-1 old adult animals carrying Tau^AT^ or the double mutant variant (Tau^AT+VM^) transgenes as extrachromosomal arrays. *Ex*[Tau^AT+VM^] shows less thrashes than the non-tg (~5 % of non-tg). But there is no difference between the single mutant variant *Ex*[Tau^AT^] and the double mutant variant *Ex*[Tau^AT+VM^]. Non-tg strain serves as control. Error bars denote SEM, n ≥ 30, ns., not significant. One-way ANOVA with Tukey’s test applied for multiple comparisons. (PDF 246 kb) Additional file 14: Mutant Tau^AT^ does not differ in phosphorylation status from wild-type Tau (Tau^wt^). Total Tau extracted from tau-tg worms was examined for the phosphorylation status at the sites known to be highly phosphorylated in the AD brain. Western blot analysis with phosphorylation specific antibodies show that tau in all the strains is highly phosphorylated at multiple sites examined (AT8 for pS199, pS202, and pT205; PHF-1 for pS396 and pS404; 12E8 for pS262 and pS356; AT180 for pT231 and pS235, AT100 for pT212 and pS214). In addition to the monomeric tau band (≈64 KDa, single arrow head), the phosphorylation specific AT8 antibody also detects a band slightly higher than 64 KDa band (double arrow head) and another band of ~120-KDa (triple arrow head) specific only to tau-transgenic worms. BV P20 = highly phosphorylated Tau of Baculovirus infected SF9 cells [79]. (PDF 355 kb) Additional file 15: Mutant tau with anti-aggregant proline substitutions (htau40A152T-I^277^P-I^308^P) localizes to the entire nervous system. Immunohistochemistry of whole worms with pan-tau K9JA antibody shows Tau^AT+PP^ localization in cell body and the neuronal processes. Panels on the left side depict nerve ring ganglion and those on the right side show ventral cord region. (PDF 174 kb)

## Abbreviations

1N4R-Tau: 412 residues, 1 insert + 4 repeats
2N4R-Tau: largest isoform of human tau in brain (441 residues, 2 inserts + 4 repeats)
ANOVA: analysis of variance
MAPT: microtubule associated protein tau
MT: microtubules
non-tg: non-transgenic
PHF: paired helical filaments
Tau^AT^: human Tau (2N4R) with mutation A152T
Tau^AT+PP^: human Tau (2N4R) with proline substitutions in the hexapeptide motifs
Tau^AT+VM^: human Tau (2N4R) with mutation A152T and V337M
Tau^AT-Nt^: N-terminal fragment of mutant A152T tau
Tau^wt^: human wild type Tau (2N4R)
Tau^wt-Nt^: N-terminal fragment of wild-type tau
Unc: uncoordinated locomotion
