# Supplementary figures and images for: Tau mutant A152T, a risk factor for FTD/PSP, induces neuronal dysfunction and reduced lifespan independently of aggregation in a C. elegans Tauopathy model
Source: Mol Neurodegener. 2016 Apr 27;11:33. doi: 10.1186/s13024-016-0096-1 (PMC4847334; doi:10.1186/s13024-016-0096-1)

Additional file 9

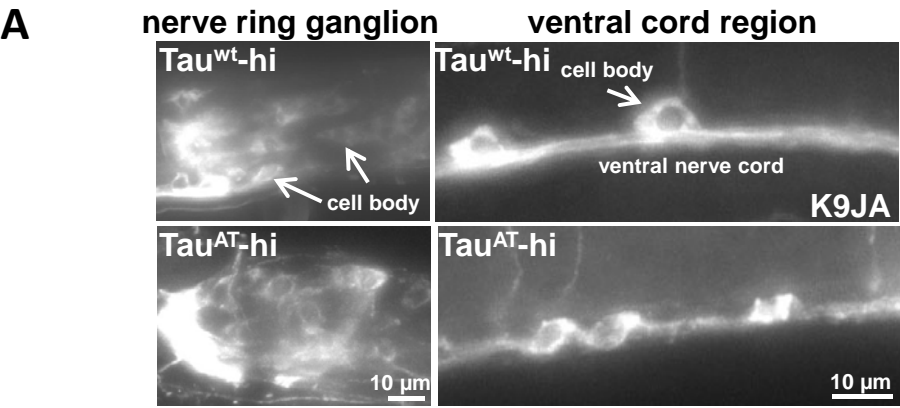

Supplement: Additional file 9: — Pan-neuronal expression of human tau in C. elegans by synaptobrevin promoter. Immunochemistry of Tauwt-hi and its mutant counterpart TauAT-hi with pan-tau antibody K9JA showing Tau staining in the nervous system. Panels on the left depict nerve ring ganglion, panels on the right side show the ventral cord region. K9JA pan-tau antibody does not stain wild-type non-tg worms (data not shown). (PDF 110 kb) [file 13024_2016_96_MOESM9_ESM.pdf]

# Additional file 10

A

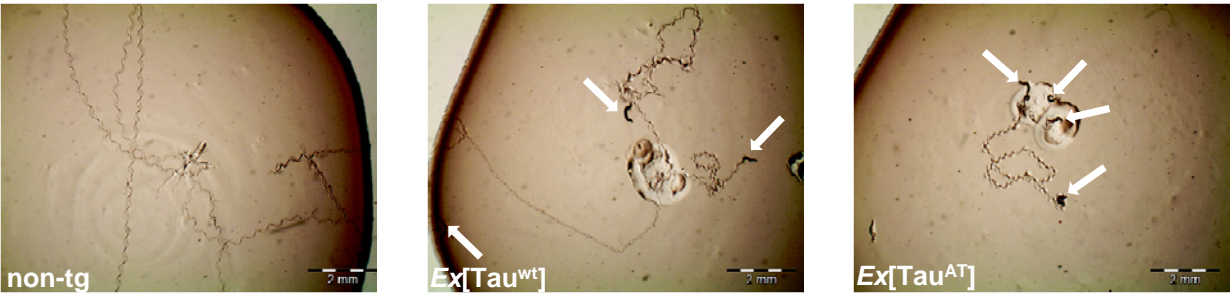

B

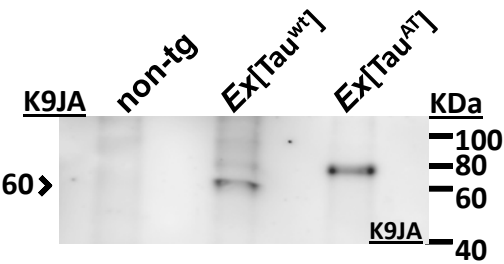

C

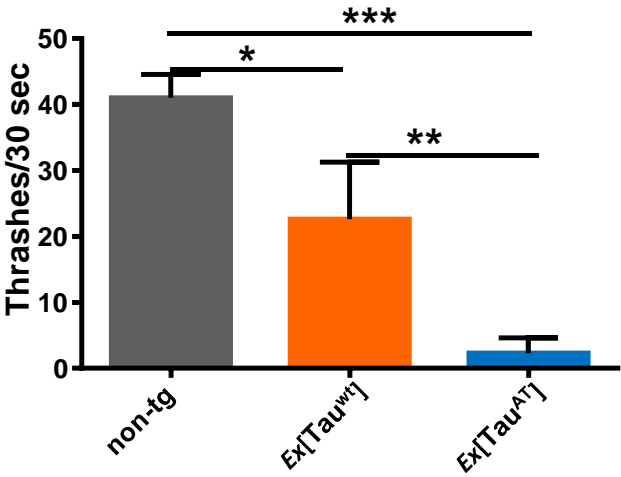

D

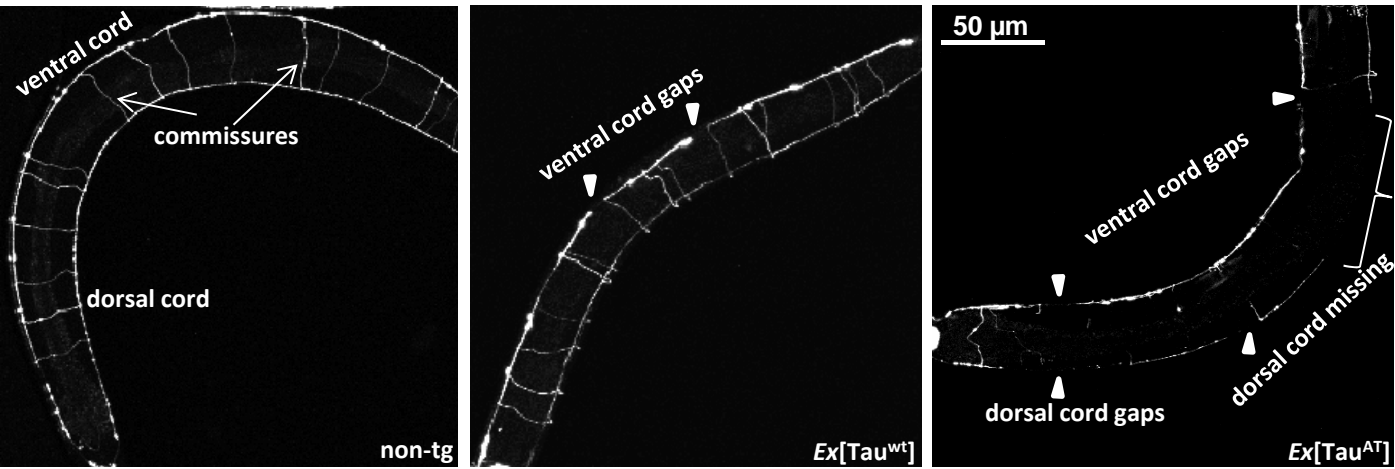

Supplement: Additional file 10: — Mutant TauAT transgene present as extrachromosomal arrays induces severe locomotion defects in C. elegans. (A) Worms carrying human tau-transgene arrays with A152T mutation Ex[TauAT] show a severe motor impairment compared to the worms carrying wild-type human tau-transgene arrays Ex[Tauwt]. Worms were allowed to crawl for 10 min after placing them onto freshly spotted NGM plates before photographing them. Ex[TauAT] worms are highly uncoordinated and show a severe paralytic phenotype obvious by the absence of tracks in the E. coli lawn and coiling behaviour. Arrows point to the displacement from the origin. (B) 30 day-1 adult animals from non-tg, Ex[Tauwt] and Ex[TauAT] lines were lysed and the lysate was subjected to 10 % PAGE and subsequent western blot analysis using K9JA-pan-tau antibody. Both Ex[Tauwt] and Ex[TauAT] carrying tau transgenes as extrachromosomal arrays show comparable expression of human tau. (C) Mean thrashing rate of day-1 old animals from strains carrying Tauwt and TauAT transgenes as extrachromosomal arrays. Non-tg wild-type strain serves as control. Error bars denote SEM, n ≥ 30. *P < 0.05, **P < 0.01, ***P < 0.001. One-way ANOVA with Tukey’s test was applied for multiple comparisons. (D) Punc-25::gfp reporter staining GABAergic motor neurons with GFP in non-tg and Tau-transgenic worms. Non-tg control worms show intact ventral and dorsal nerve cords. Transgenic worms carrying Tau-transgenes as extrachromosomal arrays show abnormal GABAergic motor neurons. Worms expressing mutant htau40A152T (Ex[TauAT]) show a severe loss of GABAergic neuronal system compared to the worms expressing wild-type tau (Ex[Tauwt]). Note the gaps (arrow heads) and absence of dorsal cord stretches (bracketed region) in Ex[TauAT] worms. (PDF 683 kb) [file 13024_2016_96_MOESM10_ESM.pdf]

## Additional file 11

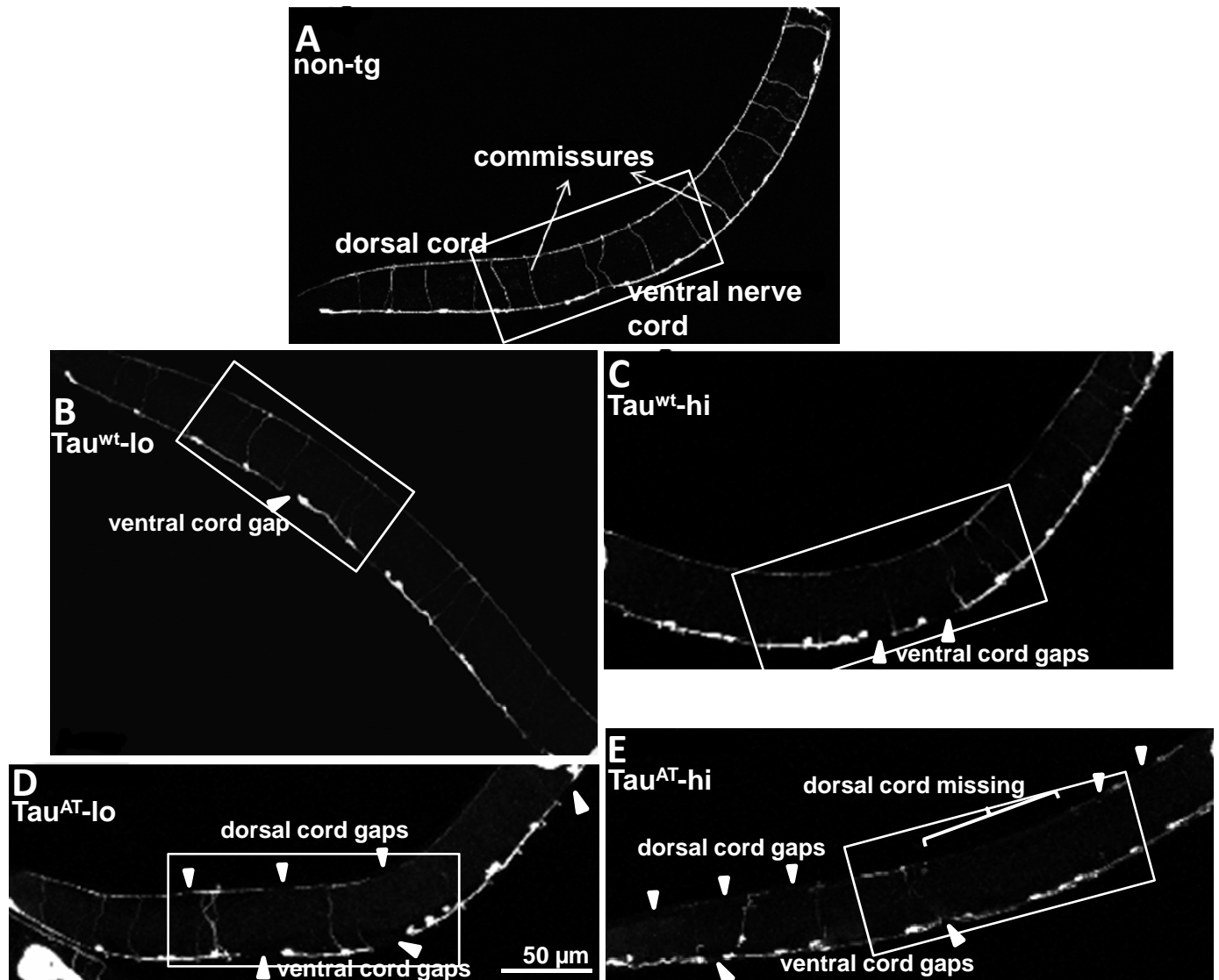

Supplement: Additional file 11: — Expression of mutant TauAT produces a substantial damage in GABAergic motor neurons. Representative whole worm maximum intensity projections (MIP) of the enlarged insets presented in main Fig. 2. Punc-25::gfp reporter that labels GABAergic inhibitory neurons with GFP, was crossed with respective tau-transgenic worms to visualize these neurons. GABAergic neurons show normal connectivity in non-tg worms, both dorsal and ventral nerve cords are intact (A). Expression of Tauwt produced dose dependent abnormalities in GABAergic neurons, with Tauwt -hi neurons (C) accumulating more damage than Tauwt -lo (B). However, mutant TauAT expression led to severe abnormalities in the form of gaps (arrowheads) in the dorsal and ventral nerve cords, in both TauAT-lo (D) and TauAT-hi (E). Also see Table 1 for detailed quantitative analysis. (PDF 276 kb) [file 13024_2016_96_MOESM11_ESM.pdf]

Additional file 12

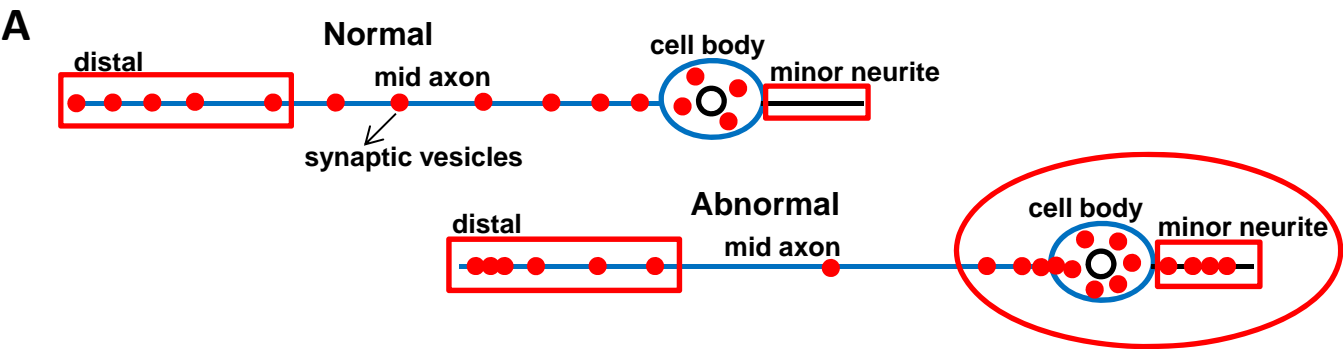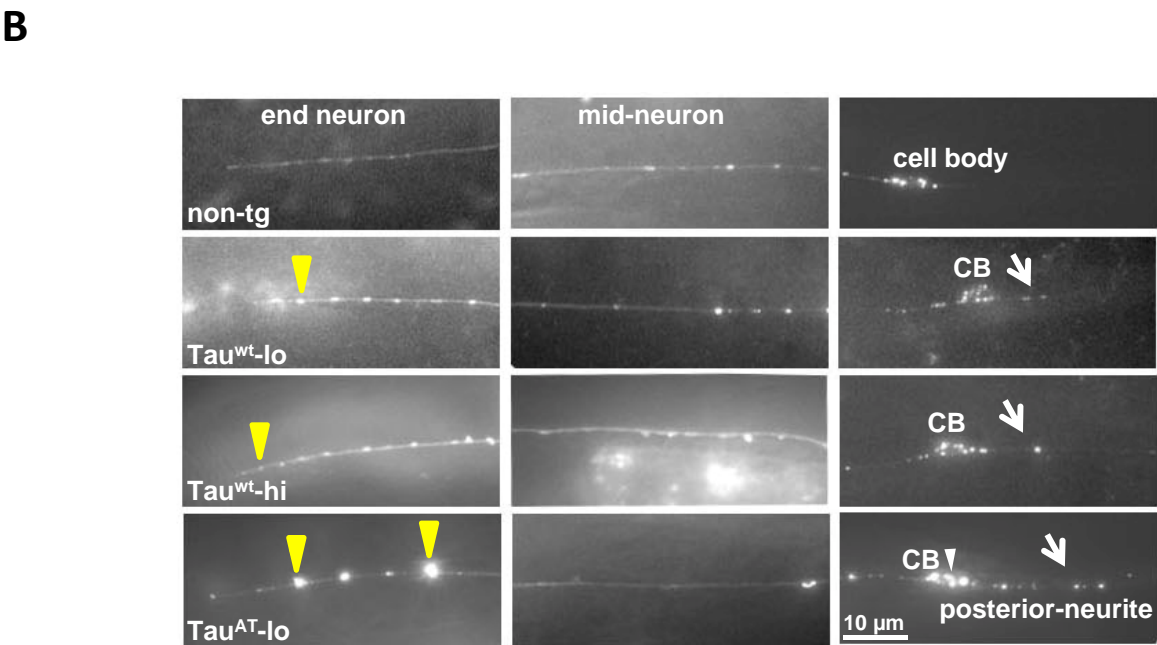

Supplement: Additional file 12: — Aberrant localization of presynaptic components in mechanosensory neurons worsens with age in mutant TauAT worms. (A) Schematic representation of presynaptic cargo distribution in a normal healthy mechanosensory neuron and in an aging neuron. (B) Presynaptic cargo visualized in day-3 old worms after crossing them into vdEx262:[Pmec-4::mCherry::rab-3] transgene, that expresses mCherry fused to synaptic vesicle associated RAB-3 in mechanosensory neurons. Mislocalization worsens in TauAT-lo, whereas Tauwt-lo and Tauwt-hi start accumulating mCherry::RAB-3 puncta in the distal axon (yellow arrowheads) and posterior neurite (white arrow). At day 3, the mid neuron of A152T worms becomes almost devoid of mCherry::RAB-3 puncta. (PDF 186 kb) [file 13024_2016_96_MOESM12_ESM.pdf]

# Additional file 13

A

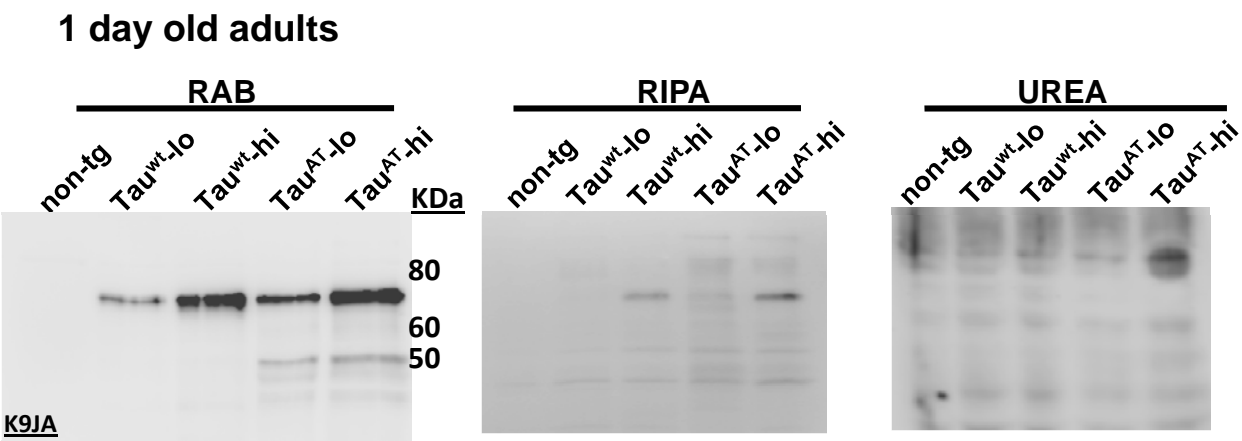

B

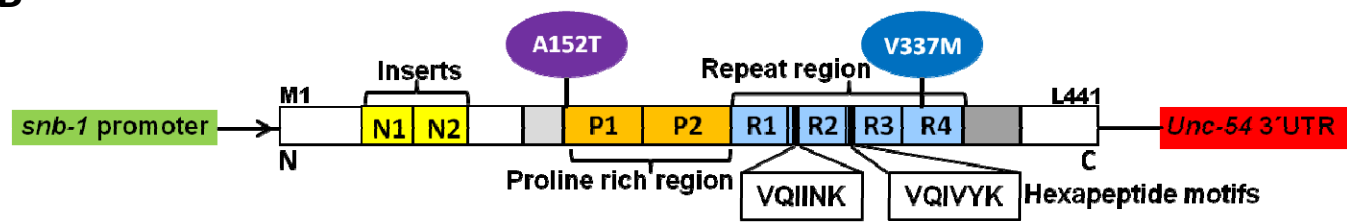

C

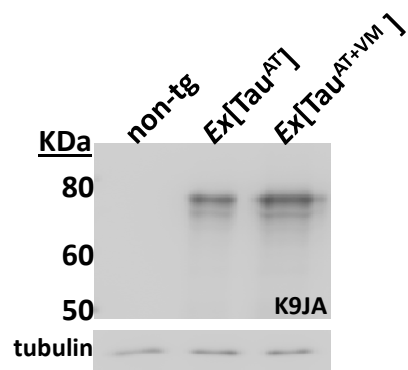

D

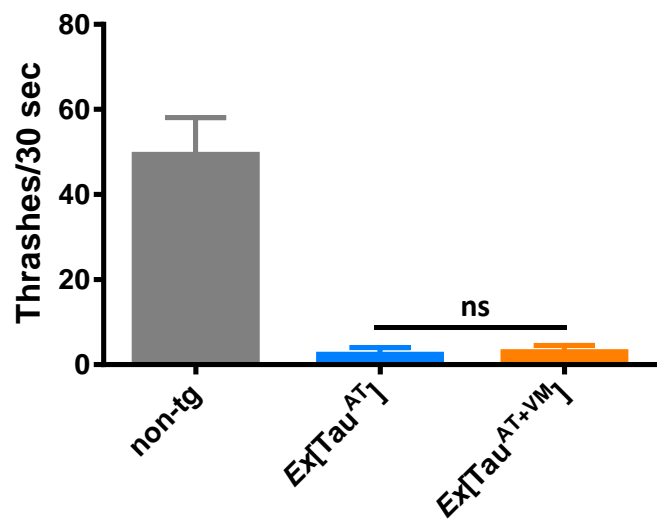

Supplement: Additional file 13: — Mutant TauAT does not aggregate in C. elegans neurons. (A) Sequential extraction reveals no insoluble tau in day-1 old adults. Tau was sequentially extracted from day-1 old transgenic worms, wild-type N2 (Bristol) worms served as control. Worms were lysed by sonication in high-salt buffer (RAB), centrifuged and supernatant collected as salt soluble Tau fraction. Reextraction of salt insoluble pellet with detergent buffer (RIPA) yielded detergent soluble Tau fraction. RIPA insoluble pellet was finally dissolved in urea buffer (UREA) to extract any insoluble fraction. Equal amount of protein was loaded and blotted using K9JA pan-tau antibody. (B) Bar diagram of Tau construct TauAT+VM with A152T mutation plus additional methionine substitution in the repeat domain (V337M) (htau40A152TV337M). (C) 30 day-1 adult animals from non-tg, Ex[TauAT] and the double mutant variant Ex[TauAT+VM] after lysis in 1 x sample buffer, then subjected to 10 % PAGE and subsequent western blot analysis using K9JA-pan-tau antibody. Ex[TauAT] and Ex[TauAT+VM] carrying the respective Tau transgenes as extrachromosomal arrays (Ex) show comparable levels of Tau expression. Tubulin serves as loading control. (D) Mean thrashing assay of day-1 old adult animals carrying TauAT or the double mutant variant (TauAT+VM) transgenes as extrachromosomal arrays. Ex[TauAT+VM] shows less thrashes than the non-tg (~5 % of non-tg). But there is no difference between the single mutant variant Ex[TauAT] and the double mutant variant Ex[TauAT+VM]. Non-tg strain serves as control. Error bars denote SEM, n ≥ 30, ns., not significant. One-way ANOVA with Tukey’s test applied for multiple comparisons. (PDF 246 kb) [file 13024_2016_96_MOESM13_ESM.pdf]

Additional file 14

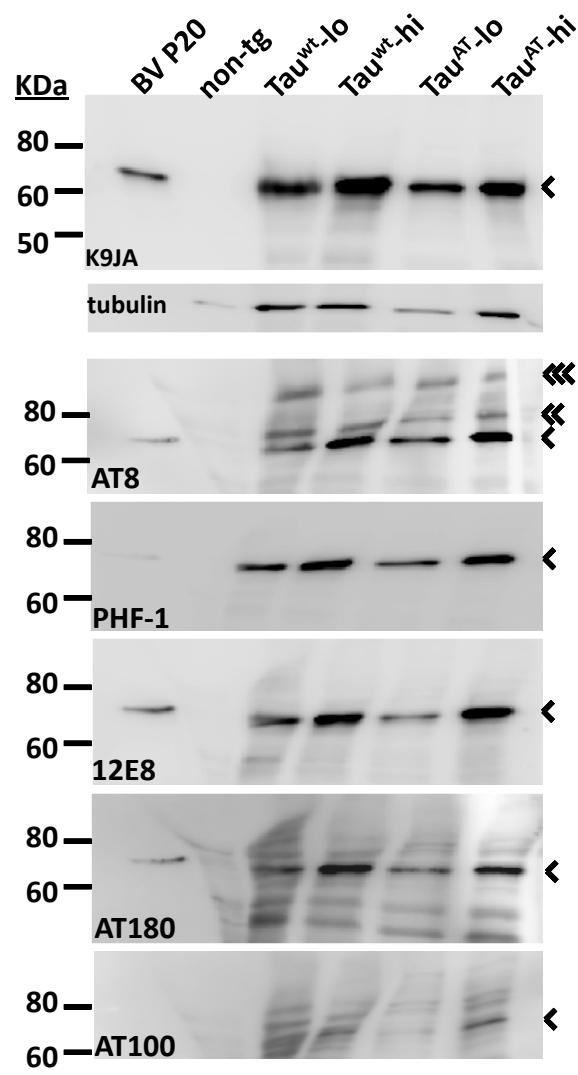

Supplement: Additional file 14: — Mutant TauAT does not differ in phosphorylation status from wild-type Tau (Tauwt). Total Tau extracted from tau-tg worms was examined for the phosphorylation status at the sites known to be highly phosphorylated in the AD brain. Western blot analysis with phosphorylation specific antibodies show that tau in all the strains is highly phosphorylated at multiple sites examined (AT8 for pS199, pS202, and pT205; PHF-1 for pS396 and pS404; 12E8 for pS262 and pS356; AT180 for pT231 and pS235, AT100 for pT212 and pS214). In addition to the monomeric tau band (≈64 KDa, single arrow head), the phosphorylation specific AT8 antibody also detects a band slightly higher than 64 KDa band (double arrow head) and another band of ~120-KDa (triple arrow head) specific only to tau-transgenic worms. BV P20 = highly phosphorylated Tau of Baculovirus infected SF9 cells [79]. (PDF 355 kb) [file 13024_2016_96_MOESM14_ESM.pdf]

Additional file 15

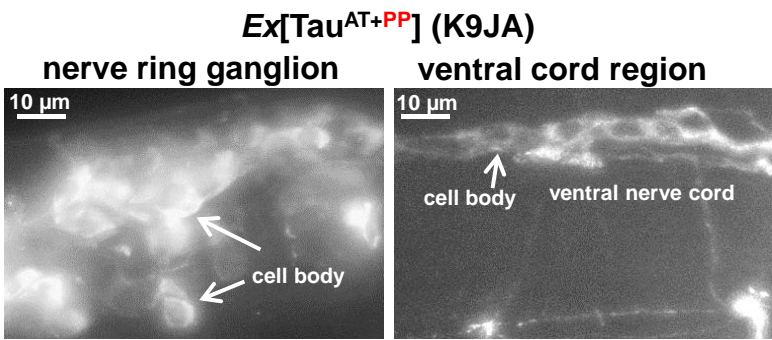

Supplement: Additional file 15: — Mutant tau with anti-aggregant proline substitutions (htau40A152T-I277P-I308P) localizes to the entire nervous system. Immunohistochemistry of whole worms with pan-tau K9JA antibody shows TauAT+PP localization in cell body and the neuronal processes. Panels on the left side depict nerve ring ganglion and those on the right side show ventral cord region. (PDF 174 kb) [file 13024_2016_96_MOESM15_ESM.pdf]
